# Exploring the bacterial diversity and its antibiotic resistance in Kabru Glacier ice cores, Sikkim Himalaya

**DOI:** 10.3389/fmicb.2025.1672943

**Published:** 2026-01-28

**Authors:** Sonia Tamang, Mingma Thundu Sherpa, Santosh Kumar, Ishfaq Nabi Najar, Prayatna Sharma, Sayak Das, Namrata Jiya, Avinash Sharma, Piyush Pandey, Nagendra Thakur

**Affiliations:** 1 Department of Microbiology, School of Life Sciences, Sikkim University, Tadong, Sikkim, India; 2 Department of Microbiology, Nar Bahadur Bhandari Government College, Tadong, Sikkim, India; 3 Department of Life Science and Bioinformatics, Har Gobind Khurana School of Life Sciences, Assam University, Silchar, Assam, India; 4 National Center for Microbial Resource, National Center for Cell Science, Pune, Maharashtra, India; 5 Department of Microbiology, Har Gobind Khurana School of Life Sciences, Assam University, Silchar, Assam, India

**Keywords:** 16S rRNA amplicon sequencing, antibiotic resistance, climate change, glacier ice core, glacier microbiome, metal tolerance

## Abstract

**Introduction:**

The Kabru Glacier, located in the Sikkim Himalayan region at an altitude of 7,318–7,412 m above sea level (a.s.l), forms part of the Kanchenjunga range in the Eastern Himalaya. Glaciers in this region are predominantly summer-fed and highly sensitive to climatic fluctuations. Despite their ecological significance, glaciers of the Sikkim Himalaya remain largely unexplored from a microbiological perspective due to harsh weather conditions and limited accessibility. In this context, the present study investigates the bacterial diversity across different depths (upper, middle, and bottom) of ice core samples collected from the Kabru Glacier.

**Methods:**

Bacterial diversity was examined using a combination of culture-dependent and culture-independent approaches. In addition, antibiotic resistance profiles and metal tolerance characteristics of the isolated bacteria were evaluated to gain further insight into their adaptive potential.

**Results and discussion:**

Culture-dependent analysis revealed a comparatively high bacterial load in the Kabru Glacier, suggesting that the biodiversity-rich Himalayan surroundings may influence the microbial community structure. Phenotypic characterization showed a predominance of Gram-positive bacteria (62.6%) over Gram-negative bacteria (37.3%). Growth profile analyses indicated optimal growth temperatures of 15°C and 20°C, with variable tolerance to salinity and pH, reflecting adaptive responses to environmental stress. Elemental analysis of ice core samples revealed higher concentrations (ppb range) of Na, Mg, K, Ca, Mn, Li, and Zn compared to other elements. Phylogenetic analysis based on 16S rRNA gene sequencing identified members of the phyla *Pseudomonadota, Bacillota*, and *Actinomycetota*. Consistently, culture-independent 16S rRNA amplicon sequencing also demonstrated the dominance of these phyla. Alpha diversity indices corroborated trends observed in the culture-dependent analysis, supporting the complementary reliability of both methodologies in elucidating bacterial community structure. Furthermore, antibiotic susceptibility testing revealed resistance to cefixime (CFM) and metronidazole (MET), along with elevated tolerance to heavy metals such as CuSO_4_, ZnCl_2_, and NiCl_2_, while showing lower tolerance to HgCl_2_.

**Conclusion:**

Collectively, these findings suggest that bacterial diversity in the Kabru Glacier is shaped by multiple environmental parameters. The occurrence of antibiotic-resistant and metal-tolerant bacteria underscores the need for further comprehensive investigations to better understand microbial adaptation and potential ecological implications in high-altitude glacial ecosystems.

## 1 Introduction

Glaciers are large masses of ice that are perennially fed by snow, creating rivers of ice. These landforms are localized in polar and other cold environments in non-polar regions (like the Himalayan range); hence, they substantially differ in their geographical location, regional climate, and local ecosystem. Apart from their scenic beauty, the major issues that need attention, such as the retreat of glaciers due to climate change, often remain unnoticed. In addition, (Pepin et al. 2015) substantiated that the rate of warming increases with elevation in high mountain regions. In glaciers, this phenomenon, known as elevation-dependent warming (EDW), is primarily driven by variability in global atmospheric circulation [e.g., El Niño-Southern Oscillation (ENSO)], snow-albedo feedback, water vapor fluxes, and aerosol deposition. The elevation-dependent warming on mountain glaciers accelerates glacier retreat, poses a risk to the glacier ecosystem, and destabilizes hydrology. Therefore, emphasis should be placed on the impact of climate change, as understanding the related risks may facilitate the prediction of future trends in climate change (Pepin et al., 2015). Since mountain glaciers (Himalayas) are regarded as the “water towers” of the world, the compounding effect of glacier retreat, in conjunction with elevation-dependent warming, significantly disrupts hydrology, increasing the possibility of global threats such as rising sea levels and Glacial Lake outburst floods (GLOF), which pose potential downstream effects for the billions of people living in the catchment area (Pepin et al., 2015; Khadka et al., 2023). Additionally, the microbial world that inhabits these freezing environments is immensely affected by the changing conditions. However, research on the microbial world of Himalayan glaciers is limited, potentially due to adverse climate and rugged terrain (Bolch et al., 2012; Rana et al., 2023).

From the beginning of the 20^th^ century, research on life beneath the glaciers commenced in the polar regions (Zhong et al., 2021; Abyzov et al., 2001; Abyzov, 1993). Ice core studies in the polar cryospheric region were established during the International Geophysical Year (IGY) of 1957–1958 (Langway, 2008). Earlier ice core research primarily focused on dating the ice, along with analyzing its elemental and isotopic composition, which provided records of past climatic conditions. Since then, ice core research has expanded and advanced across many disciplines, from isotope measurement to the study of microbes buried deep within the ice core (Jouzel and Masson-Delmotte, 2010; Liu et al., 2019). Atmospheric circulation transports microbes along with dust and elemental ions (such as Na, K, and Ca), which are commonly present in glacier ice due to atmospheric deposition and geochemical weathering. Other anthropogenic sources also contribute to their presence in the glacier through the process of wet and dry deposition (Li et al., 2024). The assessment of ions deposited in glacier ice is important, as it determines the past climatic conditions at the time of their deposition and helps identify their anthropogenic sources (He et al., 2019; Li et al., 2024). Moreover, glaciers and other snow-covered regions act as a carbon sink, holding approximately 20%−30% of the world's organic carbon (Schivalocchi et al., 2025; Brooks et al., 2011). The microbes slowly process carbon, nitrogen, and other nutrients deposited in the glaciers through biogeochemical processes, demonstrating their metabolic versatility (Simon et al., 2009). Microbial activity, mineral dissolution, and atmospheric deposition in the glacier alter the meltwater chemistry (Varliero et al., 2023). Therefore, assessing the water quality of rivers originating from glaciers is crucial, as they are the primary source of freshwater (Jadoon et al., 2025). Moreover, the melting of glaciers due to climate change can disrupt the geochemical balance, consequently changing the water composition and potentially influencing the downstream ecosystem (Xu et al., 2025). Additionally, parameters such as pH (indicating acidity/alkalinity), TDS (indicating the presence of dissolved organic and inorganic substances), and NaCl concentration (indicating osmotic balance) are key factors that shape microbial ecology (Stibal et al., 2020; Kumar and Sharma, 2021). Hence, the atmospheric particles deposited in glacier ice mainly influence the structure of the microbes inhabiting the region (Boetius et al., 2015). These archives in the glaciers provide insight into changes in climatic conditions over time (Santibáñez et al., 2018; Zhong et al., 2021).

Psychrophiles (optimum growth temperature ≤ 15 °C) and psychrotolerants (optimal growth temperature 15 °C−20 °C) are key components that constitute the bacterial community in glaciers (García-López et al., 2016; Zhong et al., 2021). However, bacterial diversity in glaciers varies according to the type of glacier, location, and regional environmental factors (García-López and Cid, 2017). Moreover, microbial communities differ along the horizontal stratification of the glacier, which is divided into three ecological zones: supraglacial, englacial, and subglacial. Environmental factors such as exposure to UV radiation, low water content, and nutrient availability overall shape the ecosystem of these glacial zones (García-López and Cid, 2017; Hotaling et al., 2017). Hence, these three strata in glaciers have distinguishable ecosystems, but the presence of liquid water channels, such as ice veins and moraines, can act as conduits for transferring abiotic particles and microbes between the different ecological zones (Hotaling et al., 2017). Although the habitats in these ecological zones are disparate, the microbial communities are not entirely confined to them. The mixing of microbial communities allows the flow of energy and nutrients along the glacial habitats, but the *in situ* activity or viability of these microorganisms in deeper regions of the glacier is questionable (Hodson et al., 2008).

Recent climate change is causing the melting of ice in glaciers, which may potentially lead to the selection of microbial communities that inhabit the cold environment. Changes in temperature and precipitation can further drive the transition of glaciers from a cold to a polythermal state, resulting in the thriving of mesophiles by superseding the existing microbial communities in the glacier habitat (García-López and Cid, 2017; Hell et al., 2013; Nowak and Hodson, 2014). As the microbial communities in the glacier are sensitive to climate change, specifically to increases in temperature, the disruption of ecological habitats (mainly due to melting of the glacier) can consequently have a significant impact on the role of these microbes in the biogeochemical cycle, metabolic potential and ultimately disrupt the community structure (García-López et al., 2019). Therefore, evaluating microbial diversity in different glacier habitats is crucial for understanding the effects of global warming (García-López et al., 2016; Zhong et al., 2021). Furthermore, microbes trapped in the ice core serve as biological proxies that provide insights into the effects of climate change, which can be used to understand past environmental conditions, and this information can also be used to predict the impacts of future climate change (Liu et al., 2024; Zhong et al., 2021). Moreover, the detection of potential pathogenic microorganisms in the ice core suggests that climate-induced melting of the glacier may release these microbes into glacier-originated river systems. Additionally, the recovery of viable and non-viable microbial populations in the ice core has been reported from polar and non-polar cryospheric regions (Christner et al., 2000; Knowlton, 2013; Priscu et al., 2007; McLean, 1918; Shen et al., 2012; Shivaji et al., 2013; Priscu et al., 1999; Zhang et al., 2003; Zhong et al., 2021). The viable bacteria obtained through culture-dependent methods demonstrate their ability to conduct metabolic activities despite the limitations imposed by various environmental factors (Liu et al., 2019). The detection of non-cultivable microbial communities in the ice core using culture-independent methods has advanced our understanding of microbial diversity at different depths within the ice core. The emergence of technologies such as 16S rRNA gene (Amplicon) sequencing has provided insight into the diversity of cold-adapted bacteria and their interactions in different habitats (Dhakar and Pandey, 2020; Paun et al., 2021). Moreover, recent advances in “omics” related technologies, which include metagenomics, transcriptomics, proteomics, and genomics, have expanded our knowledge regarding novel adaptation strategies, bacterial physiology, and the genes involved in various cellular activities (Collins and Margesin, 2019; Tribelli and López, 2018).

Culturable bacteria recovered from the ice core in the polar glacier (Christner et al., 2004; García-López et al., 2019; Miteva et al., 2015, 2004; Shivaji et al., 2013) and the non-polar glacier (Liu et al., 2019; Shen et al., 2012; Xiang et al., 2005; Zhang X. F. et al., 2008) have revealed a higher abundance of phyla belonging to *Actinomycetota, Pseudomonadota, Bacillota*, and *Bacteroidota*. However, a significant difference in the distribution of bacterial phyla was observed in these studies, depending on the location of the glacier and the depth of the ice core. Studies have found that the distribution of bacterial communities in glacier ice is not random. From the Malan Glacier and East Rongbuk Glacier, a layered distribution of bacterial communities in the ice core section was observed, indicating a bacterial response to climate change and highlighting their deposition patterns over different time periods (Xiang S. et al., 2004; Shen et al., 2012). In addition, (Christner et al. 2004) reported a higher bacterial load (~180 cfu mL^−1^) in the ice core sample from the Guliya ice cap in the Tibetan Plateau. Similarly, a higher bacterial count was observed in samples from the Yuzhufeng Glacier in the Tibetan Plateau. Although the bacterial count in the polar ice core (Vostok ice core) sample was relatively low, ranging from 10^3^ to 10^4^ CFU mL^−1^, the presence of a higher bacterial load in non-polar and Himalayan glaciers suggests the influence of rich biodiversity in the vicinity.

The Himalayas and the Tibetan Plateau are referred to as the “third pole” of the world, as they contain the largest concentration of glaciers outside the poles (Arctic and Antarctic regions) (Pant et al., 2018), and the Indian Himalayan region harbors nearly 10,000 glaciers. Hence, it is known as Asia's water tower, as the rivers originating from these glaciers sustain billions of lives in the downstream region (Kumar et al., 2020; Bajracharya et al., 2015). Although Himalayan glaciers are the least tracked for geological and microbiome studies due to the arduous topography and unpredictably harsh weather conditions (Bolch et al., 2012; Rana et al., 2023), they are of great interest to researchers, and efforts are underway to explore the unique ecosystem and biodiversity of the region. Research on the microbiome from various cryospheric regions of Himalayan glaciers has revealed psychrotrophic bacteria with the ability to produce cold-active enzymes from the Gangotri Glacier (Baghel et al., 2005; Kuddus and Ahmad, 2012); antibiotic-resistant and metal-tolerant bacterial isolates from the Changme Khang and Changme Khangpu Glacier in Sikkim Himalaya (Sherpa et al., 2020), as well as from the Hindu Kush Range Glacier (Rafiq et al., 2019), and pigment-producing psychrophilic bacteria (Kumar et al., 2022; Mukhia et al., 2024; Panwar et al., 2019; Shen et al., 2012).

The prevalence of antibiotic resistance genes (ARGs) in the natural environment suggests their presence in ancient microbiota, which evolved even before the discovery and widespread use of antibiotics in clinical settings (Segawa et al., 2013; Sherpa et al., 2020; Álvarez-Martínez et al., 2020). Antibiotic resistance has been reported from extreme environments, such as glaciers, where the impact of overwhelming anthropogenic activity is negligible (McCann et al., 2019; Morozova et al., 2022; Segawa et al., 2013; Sherpa et al., 2020; Yuan et al., 2019). In general, these antibiotic resistance genes are commonly disseminated in mesophilic bacteria via horizontal gene transfer (HGT) through extrachromosomal DNA elements, including plasmids and integrons (Orlek et al., 2023; Amarasiri et al., 2020). The occurrence of antibiotic resistance genes in cold-loving bacteria in glaciers may suggest their transmission through aeolian processes and the excrement of migratory birds (Segawa et al., 2013; Sherpa et al., 2020). Another important aspect that requires attention is the presence of heavy metals in glaciers, which are inorganic pollutants originating from anthropogenic activities and are deposited in pristine environments primarily through atmospheric circulation (Sherpa et al., 2020). It is crucial to study heavy metal contamination in glacial environments because these compounds are persistent and non-biodegradable, and even at low concentrations, they are toxic (Biswas et al., 2021). These recalcitrants may be released into the water system due to the melting of glaciers in response to climate change, potentially contaminating glacier-fed rivers (Suyal et al., 2021). In a metal-contaminated environment, bacteria acquire a metal resistance system in response to various selective pressures (Benhalima et al., 2020). These metal-tolerating bacteria are essential for the bioremediation of contaminated sites, providing an effective alternative to chemical treatment methods. Furthermore, understanding the adaptive nature of metal-tolerant bacteria in cold environments, such as glaciers, is crucial for their potential use in bioremediation in these areas (Biswas et al., 2021; Suyal et al., 2021; Pittino et al., 2023). Recovery of metal-tolerating bacterial isolates has been reported from the ecological sites of the Hindu Kush Himalayan Glaciers (Rafiq et al., 2019) and from our previous study conducted on two glaciers of Sikkim Himalaya (Changme Khang and Changme Khangpu Glacier), which also showcased the prevalence of metal resistance genes (MRGs) in the bacterial population of the glacier ice sample (Sherpa et al., 2020).

Studies have accentuated the co-existence of antibiotic resistance genes (ARGs) and metal resistance genes (MRGs) in bacteria (Di Cesare et al., 2016; Sherpa et al., 2020), highlighting the importance of understanding the co-selection pattern in bacterial populations harboring different resistance genes (Di Cesare et al., 2016; Murray et al., 2024). Co-selection refers to the concomitant selection of different resistance genes, such as metal and antibiotic resistance genes, where the presence of one selective agent (e.g., heavy metal) allows a microorganism to acquire not only metal resistance but also promotes the acquisition of antibiotic resistance genes (Di Cesare et al., 2016; Gillieatt and Coleman, 2024). The process of co-selection can occur through three genetic mechanisms: co-resistance (which occurs when multiple resistance genes are physically linked in the same mobile genetic element, resulting in simultaneous selection), cross-resistance (which occurs when a resistance mechanism [e.g., efflux pump] confers resistance to both metals and antibiotics), and co-regulation (which occurs when the presence of one stressor [e.g., heavy metal] leads to the activation of regulatory events that trigger the expression of both antibiotic resistance genes and metal resistance genes) (Kumar et al., 2023; Murray et al., 2024; Gillieatt and Coleman, 2024). Therefore, heavy metals are non-degradable stressors that potentially promote the dissemination of antibiotic resistance genes (Baker-Austin et al., 2006). However, in the glacier environment, an intricate investigation is necessary regarding the co-selection mechanisms in bacteria buried in glacier ice, as the melting of glaciers due to global warming may release these bacteria carrying resistant genes into downstream waterways (Sherpa et al., 2020).

Sikkim Himalaya, which is a part of the eastern Himalayan range, hosts nearly 84 glaciers (Bahuguna et al., 2001). These high mountain glaciers are potentially more affected by elevation-dependent warming due to climate change than the lower-elevation region (Pepin et al., 2015). Research on the glaciers of the Sikkim Himalaya is significant for understanding the consequences of climate change, as the increase in temperature in the high-altitude glacier environment has a substantial impact on its ecosystem, regional hydrology, and indigenous microbial communities. Moreover, studies on the microbiological aspects of glaciers in this range are few. Although a few studies are available regarding the microbiological findings from the sources of the Sikkim Himalayan glaciers, to date, no studies have been conducted in the ice core region of the glaciers in the Sikkim Himalayas. Therefore, this study is unique, as it highlights the bacterial diversity in an ice core sample from the Kabru Glacier, located in the western part of the Sikkim Himalayan range. It provides a comparative analysis of bacterial diversity at different depths using both culture-dependent and culture-independent methods (16S rRNA gene amplicon sequencing). The assessment of the metabolic potential of the glacier isolates provides clues to their adaptive mechanisms, which may support a cross-resistance mechanism that offers protection against heavy metals and antimicrobial agents. Hence, we have studied the physiological (growth profile) and biochemical characteristics of the isolates from the glacier ice. Additionally, this research has examined the quantitative analysis of the isolates for antibiotic resistance and their potential to tolerate metals.

## 2 Materials and methods

### 2.1 Description of sampling site

Kabru Glacier, which originates between 7,318 and 7,412 m a.m.s.l. (meters above mean sea level), is an important glacier of the Sikkim Himalaya, situated in West Sikkim, India. It lies on the southern flank of the Kanchenjunga massif in the Eastern Himalaya. The glacier flows down from the accumulation zone through steep, rugged terrain and forms one of the headwater sources of the Teesta River system, which is vital for the hydrology of Sikkim. The glacier is predominantly summer-fed, receiving input from monsoonal precipitation and seasonal snow accumulation (Hasnain, 2002; Thayyen and Gergan, 2010). This makes it highly sensitive to fluctuations in temperature and precipitation patterns. Kabru Glacier also represents an ecologically fragile zone, supporting specialized microbial life, cold-adapted flora, and unique alpine habitats. Its meltwater contributes not only to sustaining river flow but also to regulating nutrient cycles in the high-altitude catchments.

### 2.2 Sample collection

**Figure 1:**
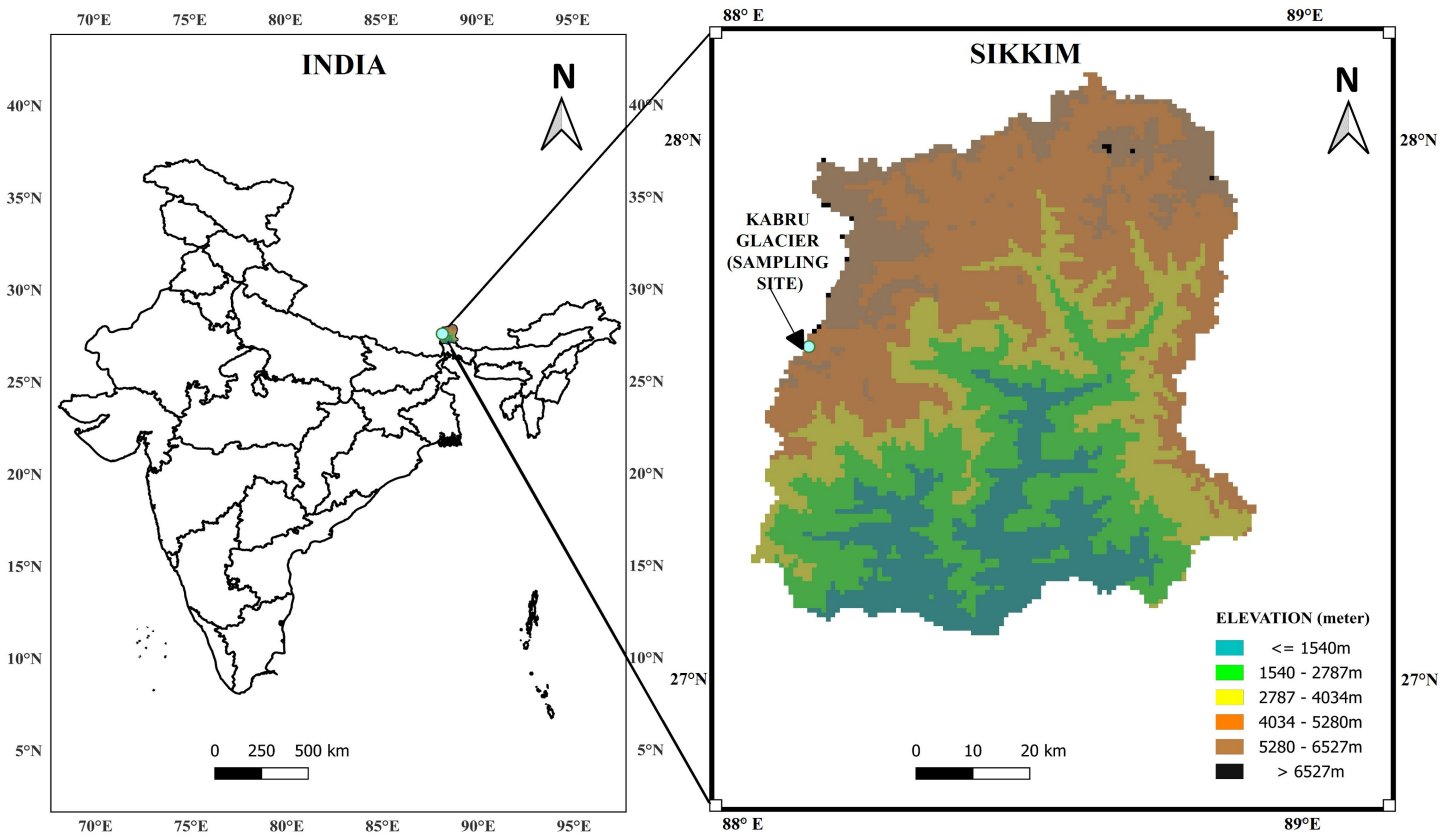
Map of Kabru Glacier sampling sites in Sikkim, India, with coordinates obtained via Global Positioning System (GPS78SMAP) and mapped using QGIS (v3.42.3).

Ice core samples were collected from the accumulation zone of Kabru Glacier (Sikkim, India) in October 2021. The geographic coordinates and elevation of the sampling sites were determined using a handheld GPS device (GPSMAP 78S, Garmin, India) (Table 1). Site mapping was conducted using QGIS software (version 3.42.3) (Figure 1). The radiant surface temperature at the sampling location was measured using a non-contact infrared thermometer (Laser IR Point Digital LCD, Taiwan, China). Approximately 1.5 m of ice core was extracted using a portable ice core drilling machine (KOVACS Enterprise, USA), which retrieves a 0.15 m diameter ice core with a 1.5 m long core barrel. The retrieved ice core sample was divided into three sections (approximately 0.5 m each) using a sterile saw-tooth knife: upper (0–0.5 m), middle (0.5–1.0 m), and bottom (1.0–1.5 m). The collected cores were placed in sterile Cello chiller ice boxes (Cello, Mumbai, India) and transported to the laboratory under aseptic conditions. In the laboratory, all processing was performed at a temperature below 20 °C in a positive-pressure laminar airflow hood to maintain sterility (Zhang et al., 2003). The outer 5 mm annulus of each section (upper, middle, and bottom core) was removed with a sterile saw-tooth knife. The remaining inner core was surface sterilized with chilled 95% ethanol and rinsed with cold (4 °C) autoclaved distilled water (Sherpa et al., 2018b; Xiang S. R. et al., 2004; Zhang et al., 2003). Finally, the sterile core sections were transferred to Borosil glass beakers (Borosil, India) and allowed to melt at 4 °C in an incubator prior to further analysis. Approximately 1,500 ml of meltwater was retrieved from each ice core sample and was allocated for culture-dependent and culture-independent studies. In brief, out of 1,500 ml of meltwater, 50 ml was allocated for physicochemical analysis (10 ml for ICP-MS and 40 ml for analysis of physical parameters). For DNA extraction intended for 16S rRNA gene (amplicon) sequencing, 500 ml of meltwater was processed to ensure adequate biomass recovery, given the low microbial load in the glacier meltwater. From the remaining volume (~950 ml), 450 ml of meltwater was used for a culture-dependent study, while 500 ml was reserved for additional studies relevant to glacier microbial ecology. For the negative control, autoclaved distilled water filtered through 0.22 μm syringe-driven filters (HiMedia, Mumbai, India) was used. The background controls (field and melt blanks) were processed identically to the ice samples to systematically assess the contaminants introduced during sample handling.

**Table 1:** Physical parameters and microbial counts at different depths of the ice core sample retrieved from the Kabru Glacier.

**Sample**	**CB1 (upper core)**	**CB2 (middle core)**	**CB3 (bottom core)**
Coordinates	27.63455N/88.11657E		
Elevation	4,918 m a.s.l.	4,918 m a.s.l.	4,918 m a.s.l.
Temperature	−21 °C	−21 °C	−21 °C
TDS (g L^−1^)	11	9.5	3.4
pH	6.4	6.6	6.5
NaCl (ppb)	14	12	10
Microbial count (CFU mL^−1^)	3.5 × 10^8^	5.9 × 10^6^	7.0 × 10^8^

Microbial counts represent culturable bacteria obtained after enriching ice core samples; *in situ* abundance remains unknown.

### 2.3 Physicochemical analysis

Physical parameters of the samples, including pH, total dissolved solids (TDS), and NaCl concentration, were measured in the laboratory using a portable water quality checker (Horiba U-50 Series, Japan). The samples were then passed through 0.22 μm syringe-driven filters (HiMedia, Mumbai, India) to remove particulates. Elemental analysis was performed using inductively coupled plasma mass spectrometry (ICP-MS) with an Agilent 7,900 instrument (Agilent Technologies, USA). A total of 19 elements were quantified, and their concentrations (in ppb) were recorded for subsequent evaluation.

### 2.4 Culture-dependent study

#### 2.4.1 Enrichment-dependent isolation

To enhance bacterial recovery, 10 ml of ice core meltwater (from each ice core sample) was enriched in four different media: Luria Bertani broth (HiMedia, Mumbai, India), Antarctic Bacterial Medium (HiMedia, Mumbai, India), R2A (HiMedia, Mumbai, India), and King's B broth (HiMedia, Mumbai, India) in a 250 ml conical flask (Borosil, India). Incubation was carried out at 15 ± 1 °C in a BOD shaking incubator for 1 week at 120 rpm. The enriched cultures were plated onto the respective media (in triplicate) and incubated at 15 °C for 120 h (5 days). Distinct bacterial colonies were selected based on their colony morphology, and pure colonies were then isolated and subcultured in Luria–Bertani medium (HiMedia, Mumbai, India) to ensure optimal growth and viability of the bacterial cultures. The bacterial load (CFU mL^−1^) was quantified from the enrichment cultures of each ice core meltwater sample by plating onto Luria Bertani (LB) agar. Therefore, the CFU counts represent the post-enrichment, culturable bacterial representatives. For long-term preservation, cultures were mixed with sterile glycerol to a final concentration of 50% (v/v) and stored at −80 °C until further use (Najar et al., 2018; Rafiq et al., 2017).

#### 2.4.2 Phenotypic characterization

Bacterial colonies with distinct morphological characteristics were recorded. To further differentiate the isolates, a series of biochemical tests was performed to assess their metabolic properties. These included carbohydrate fermentation, nitrate reduction, indole production, citrate utilization, the methyl red test, and the Voges–Proskauer test. In addition, enzymatic screening for amylase and protease activity was conducted to assess the ability of the isolates to hydrolyse starch and casein, respectively (Ottoni et al., 2020; Salwan et al., 2010). To minimize redundancy among isolates showing similar 16S rRNA gene sequences and phenotypic traits, a total of 10 bacterial isolates from each ice core were selected based on distinct morphological and biochemical characteristics for further study.

#### 2.4.3 Growth profile at various physical parameters

To determine the optimum temperature range for bacterial growth, the isolates were incubated at 10 °C, 15 °C, 30 °C, 37 °C, and 50 °C in a shaker incubator for 120 h. After incubation, growth was assessed by measuring the optical density (OD) of the broth cultures at 600 nm using a UV–Vis spectrophotometer (PerkinElmer LAMBDA 40, USA). Salt tolerance was evaluated by culturing the isolates in Luria Bertani (LB) broth supplemented with NaCl at concentrations of 0.2%, 0.5%, 1%, 5%, and 10% (w/v) (Rafiq et al., 2017). Following incubation in a cooling incubator at 15 °C for 120 h, the OD of each culture was measured at 600 nm to determine the effect of salinity on growth. For pH tolerance, the isolates were inoculated into media adjusted to pH values of 3.0, 5.0, 7.0, 9.0, and 11. Cultures were incubated at 15 °C in a cooling incubator for 120 h, and growth was quantified by measuring the OD at 600 nm after incubation.

#### 2.4.4 Antibiotic resistance profiles of glacier isolates

The antibiotic susceptibility of the bacterial isolates was tested using the Kirby–Bauer disc diffusion method (Kumar et al., 2023; Hudzicki, 2009). Standardization of the assay was carried out according to the CLSI guidelines (Humphries et al., 2023). The optical density (OD) of the overnight broth culture of the respective isolate was adjusted to a 0.5 McFarland standard, and 0.1 mL of each culture was evenly swabbed onto Mueller–Hinton Agar (MHA) plates using sterile cotton swabs. Sterile antibiotic discs were then placed on the inoculated plates, while sterile membrane filter paper discs served as negative controls. A total of 16 antibiotics belonging to seven different classes were tested. The isolates were screened against Ampicillin (AMP, 10 μg), Chloramphenicol (C, 30 μg), Erythromycin (E, 15 μg), Methicillin (MET, 10 μg), Streptomycin (S, 100 μg), Vancomycin (VA, 30 μg), Tetracycline (TET, 30 μg), Ciprofloxacin (CIP, 5 μg), Gentamicin (GEN, 30 μg), Doxycycline Hydrochloride (DO, 10 μg), Nalidixic Acid (NA, 30 μg), Cefixime (CFM, 5 μg), Ofloxacin (OF, 5 μg), Imipenem (IMP, 10 μg), Amoxicillin (AMC, 30 μg), and Azithromycin (AZM, 15 μg). After incubation, the diameter of the inhibition zone was measured (in mm) and interpreted by comparing it with the zone diameters established for Gram-negative (*Enterobacter*) and Gram-positive (*Staphylococcus*) genera, as per CLSI guidelines (Sherpa et al., 2020). The Multiple Antibiotic Resistance (MAR) index was calculated for each isolate using the formula:


$$\begin{array}{l} \text{ Multiple Antibiotic Resistance }(\text{MAR})\text{ index }=\\ \frac{\text{Number of antibiotics to which the isolates were resistant}}{\text{Number of tested antibiotics}} \end{array}$$

#### 2.4.5 Determination of minimum inhibitory concentration assay

The MIC of selected antibiotics against Kabru Glacier isolates was determined using the microbroth dilution method. Four groups of seven classes of antibiotics were tested: β-lactams [ampicillin, methicillin, amoxicillin, and cefixime (cephalosporin)], aminoglycosides (streptomycin), glycopeptides (vancomycin), and macrolides (erythromycin). Antibiotic stock solutions were serially diluted to obtain concentrations ranging from 0.1 to 16 mg/L. For each assay, 50 μL of sterile Mueller–Hinton broth (HiMedia, Mumbai, India) was dispensed into the wells of a microtiter plate, followed by the addition of antibiotics at different concentrations (0.1–5 mg/L, serially diluted 2-fold). Each well was then inoculated with 50 μL of bacterial suspension adjusted to OD600 ≈ 0.1 (corresponding to ~5 × 10^5^ CFU mL^−1^). Negative controls consisted of broth without antibiotics. Plates were incubated at 15 °C for 120 h in a cooling incubator. After incubation, 30 μL of resazurin solution (0.015%) was added to each well, and the plates were further incubated for 2–4 h. Wells that remained blue (no color change of resazurin) were recorded as exceeding the MIC value (Elshikh et al., 2016; Feßler et al., 2023). The MIC values obtained were compared with the breakpoint standards provided by CLSI guidelines. Since no standardized breakpoints are available for psychrophilic or psychrotolerant bacteria, interpretive criteria for psychrotolerant genera such as *Pseudomonas* and *Enterobacter* were applied.

#### 2.4.6 Heavy metal tolerance assay

The selected isolates were screened for heavy metal tolerance against five analytical-grade heavy metal salts: ZnCl_2_, CuSO_4_, NiCl_2_, CoCl_2_, and HgCl_2_. Stock solutions of 0.01 M, 0.1 M, and 1 M were prepared in distilled water and filter-sterilized (Dziewit et al., 2013). The microbroth dilution method was employed to assess heavy metal tolerance. Serial dilutions of the sterile stock solutions (ranging from 0.001 M to 0.5 M, with 2-fold dilution steps) were prepared in Luria–Bertani (LB) broth within 96-well microtiter plates. Each well was inoculated with 50 μL of a bacterial suspension adjusted to OD600 ≈ 0.1 (approximately 5 × 10^5^ CFU mL^−1^). Wells containing only LB broth without heavy metals served as negative controls. Plates were incubated at 15 °C for 120 h in a cooling incubator, and bacterial growth was subsequently monitored using an automated microplate reader (Ciok et al., 2018; Dziewit et al., 2013).

#### 2.4.7 Genotypic characterization

Genomic DNA from the bacterial isolates was extracted using the QIAamp DNA Mini Kit (QIAGEN, India) following the manufacturer's protocol. The 16S rRNA gene was amplified by PCR using universal primers 27F (5′-AGAGTTTGATCCTGGCTCAG-3′) and 1492R (5′-CGGTTACCTTGTTACGACTT-3′) (Lane, 1991; Frank et al., 2008). PCR amplification was performed in a Bio-Rad thermal cycler under the following conditions: initial denaturation at 94 °C for 5 min, followed by 30 cycles of denaturation at 94 °C for 1 min, annealing at 55 °C for 1 min, and extension at 72 °C for 1.5 min. A final extension was carried out at 72 °C for 10 min. The quantity of extracted DNA was measured using a Qubit Fluorometer (Thermo Fisher Scientific, USA), and quality was assessed by electrophoresis on a 0.8% agarose gel (Najar et al., 2020). PCR products were sequenced bidirectionally using primers 27F and 1492R on an ABI 3500 Genetic Analyzer (Applied Biosystems, USA). The raw ABI sequence files were visualized and assembled using Finch TV version 1.4.0 (Kisand and Lettieri, 2013) (Geospiza Inc. https://finchtv.software.informer.com/1.4/). Assembled sequences were compared against reference sequences in the GenBank database using the BLASTn tool (Mizrachi et al., 2007) (NCBI; http://blast.ncbi.nlm.nih.gov/Blast.cgi), with a taxonomic assignment threshold of ≥97% sequence similarity. The phylogenetic relationships of the identified isolates were inferred using the neighbor-joining method in MEGA version 12.1 software (Saitou and Nei, 1987). The finalized 16S rRNA sequences were submitted to the NCBI GenBank for accession number assignment.

## 3 Culture-independent study

### 3.1 Genomic DNA extraction and purification

Total genomic DNA was extracted from the melted ice samples using the DNeasy PowerWater Kit (MO BIO Laboratories, USA) following the manufacturer's instructions. The quantity of extracted DNA was measured using a Qubit Fluorometer (Thermo Fisher Scientific, USA), and the quality was assessed by electrophoresis on a 0.8% agarose gel (Najar et al., 2020).

### 3.2 Library preparation for 16S rRNA amplicon sequencing

The extracted DNA was amplified by targeting the V4 hypervariable region of the 16S rRNA gene using universal primers 515F (5′-GTGCCAGCMGCCGCGGTAA-3′) and 806R (5′-GGACTACHVGGGTWTCTAAT-3′) (Caporaso et al., 2011). To reduce amplification bias and enhance reproducibility, the DNA sample was subjected to PCR reactions in triplicate, which were pooled prior to library preparation. Amplicon library preparation was performed according to the manufacturer's 16S Metagenomics protocol (Illumina, USA). Equimolar concentrations of the 16S rRNA amplicon libraries were pooled and loaded onto the MiSeq system for sequencing, using a paired-end read length of 2 × 250 bp (Jani et al., 2018). The negative controls underwent the same library preparation and sequencing protocol. The sequences generated were submitted to the NCBI BioProject database under BioProject ID PRJNA1109674, where the Sequence Read Archive (SRA) run accession numbers associated with this study are SRR28976176 (Upper Core), SRR28976175 (Middle Core), and SRR28976164 (Bottom Core).

### 3.3 Sequence quality control and bioinformatics analysis of amplicon sequence

The quality assessment of the sequences was executed with the FastQC program, and the individual report from FastQC was compiled using MultiQC (v1.10.1) (Ewels et al., 2016). The adapter-removed sequences with a Phred quality score greater than 30 and a read length greater than 100 bp were retained and further subjected to pre-processing steps. The paired-end read library was assembled using MEGAHIT (v1.2.9) (Li et al., 2015). Demultiplexing, sequence filtering, and removal of chimeric sequences were achieved using the built-in tools in the LotuS2 pipeline (https://usegalaxy.eu/) (Özkurt et al., 2022). DADA2 (implemented with default parameters in the LotuS2 pipeline) was used for ASV clustering of the high-quality reads. DADA2 collapses redundant sequences within the high-quality reads and infers unique amplicon sequence variants (ASVs) (Callahan et al., 2016). The taxonomic assignment of the ASVs, such as phylum, order, family, genus, and species, was performed using the LAMBDA aligner with the LCA algorithm (within the LotuS2 pipeline) (Hauswedell et al., 2014), against the SILVA 138.2 reference database (default within LotuS2) (Yilmaz et al., 2014). Additionally, downstream analyses of the reads—alpha and beta diversity estimation, and correlation analysis of the top 20 most abundant genera among different variables—were computed using the R package (Packages used for creating heatmaps: pheatmap) (Kolde and Kolde, 2015), tidyverse, vegan, RColorBrewer; PCA plot: ggplot2, factoextra, FactoMiner, tidyr, RColorBrewer; alpha diversity: dot plot with *geom_jitter*, ggplot2.

## 4 Results

### 4.1 Physicochemical properties of glacier samples

The analysis of the physical parameters of the ice core samples revealed that the TDS (g L^−1^) and NaCl (ppb) concentrations were higher in the upper section of the Kabru Glacier core. The pH of the melted ice samples was found to be slightly acidic in all three cores—upper, middle, and bottom (Table 1). The examination of chemical parameters revealed that, among a total of 19 elements, most were present at lower concentrations compared to those found in other environments. However, elements such as Na, Mg, K, Ca, Mn, Li, and Zn were detected at higher concentrations (in parts per billion, ppb) relative to the other elements (Table 2).

**Table 2:** Elemental composition of three ice core samples from Kabru Glacier.

**Sample name**	**CB1**	**CB2**	**CB3**
Li	1.105	0.357	0.260
Na	113.359	145.517	85.228
Mg	41.862	14.740	12.916
P	0	0	0
K	154.865	90.212	57.972
Ca	51.948	17.669	15.549
Cr	0	0.017	0
Mn	7.013	3.963	2.406
Fe	0	0	0
Co	0.312	0.076	0.057
Ni	1.562	1.833	0.866
Cu	0.003	0	0
Zn	14.185	9.522	7.784
As	0	0.047	0.016
Se	0.132	0	0.264
Mo	0.003	0.006	0
Ag	0	0	0
Pb	0	0	0
Cd	0.170	0.054	0.263

The elemental concentration was measured in parts per billion (ppb).

### 4.2 Culture-dependent

#### 4.2.1 Isolation and characterization of bacterial isolates

The culturable bacterial count (culturable bacterial representatives recovered from the enrichment of ice core meltwater) ranged from 5.9 × 10^6^ to 7.0 × 10^8^ CFU mL^−1^. Core-wise analysis revealed that the bottom core (CB3) harbored the highest bacterial load (1.95–7 × 10^8^ CFU mL^−1^; mean 4.47 × 10^8^ CFU mL^−1^), followed by the upper core (CB1) (1.38–3.5 × 10^8^ CFU mL^−1^; mean 2.44 × 10^8^ CFU mL^−1^) and the middle core (CB2) (1.12–5.9 × 10^6^ CFU mL^−1^; mean 3.51 × 10^6^ CFU mL^−1^) (Table 1). However, the CFU counts represent post-enrichment growth outcomes and do not directly measure the actual *in situ* bacterial abundance. Gram reaction analysis showed a predominance of Gram-positive bacteria (62.66%) over Gram-negative bacteria (37.33%). Notably, Gram-negative bacteria were more abundant in the middle core (CB2), whereas Gram-positive bacteria dominated the bottom core (CB3), followed by the upper core (CB1). Morphologically, most isolates were white and rod-shaped, with a few being cocci-shaped. Biochemical tests indicated that most middle core (CB2) isolates were positive for citrate and nitrate reduction, whereas bottom core (CB3) isolates were positive for the indole test; eight isolates tested negative for citrate and Methyl Red-Voges Proskauer (MR-VP) tests (Supplementary Table 1). Carbohydrate fermentation assays revealed that upper core isolates could ferment most of the tested sugars, followed by bottom core isolates, while middle core isolates fermented only dextrose, galactose, and arabinose (Supplementary Table 2). Amylase and protease activities of the isolates are summarized in Supplementary Table 3.

#### 4.2.2 Growth pattern at different temperatures, pH and NaCl

The growth profile of the bacterial isolates from the ice core samples was examined. For the majority of isolates, growth tolerance was observed between 10 °C and 37 °C, indicating that they were cold-tolerant bacteria. The optimum growth temperature, as recorded after 120 h of incubation, was 15 °C and 20 °C. Growth under different NaCl concentrations (w/v) showed that most isolates grew best at 1% NaCl. Although some isolates tolerated up to 5% NaCl, the majority exhibited adequate growth at ≤ 1% NaCl. Growth was also evaluated across a pH range of 3–11. The isolates showed optimum growth between pH 7 and 9, with some demonstrating tolerance and prominent growth above pH 5 (Supplementary Table 4).

#### 4.2.3 Identification of bacterial isolates and phylogeny

**Figure 2:**
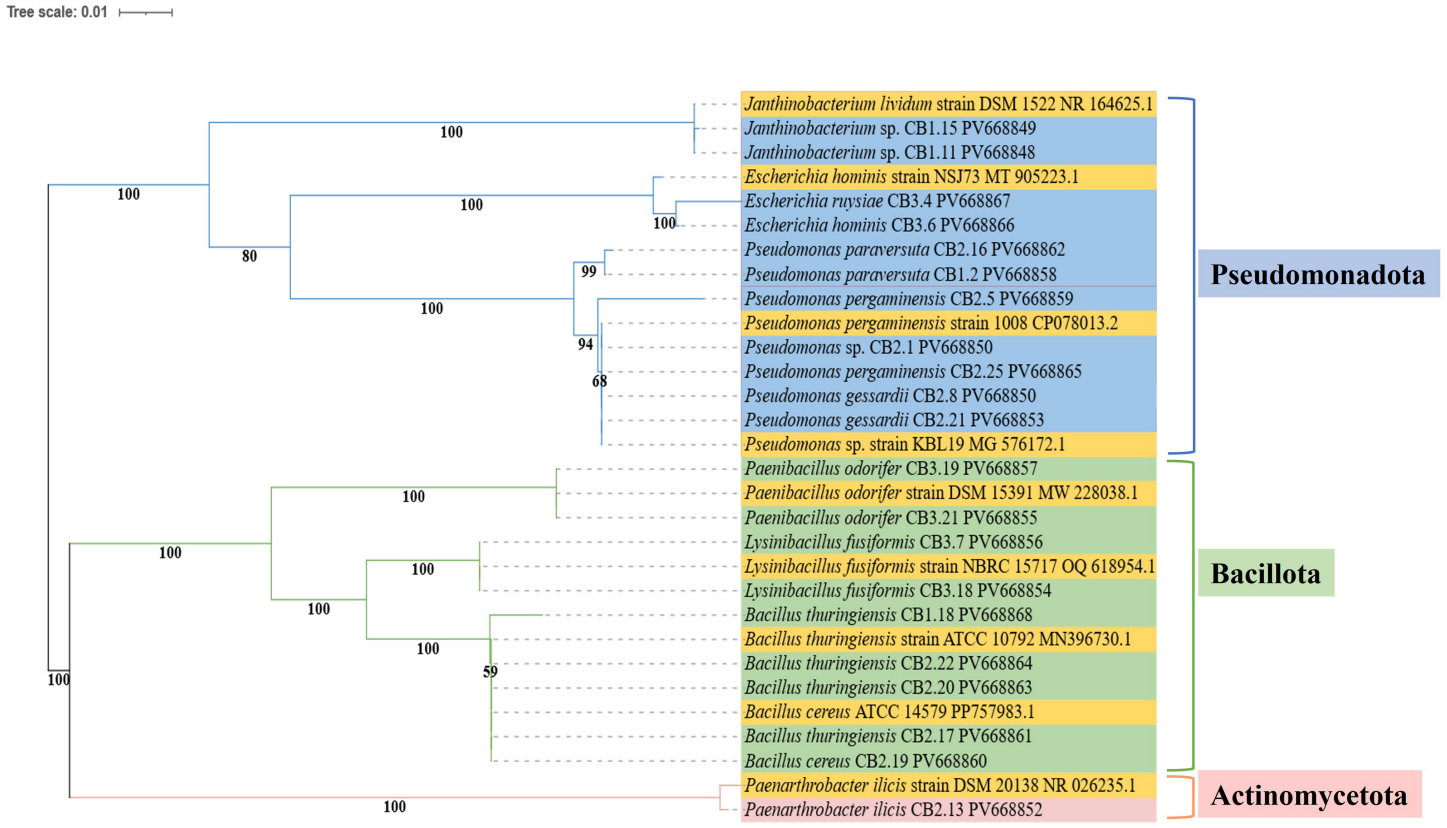
The phylogenetic tree of 16S rRNA gene sequences from Kabru Glacier isolates, constructed using the neighbor-joining method (Jukes-Cantor model) in MEGA 12, validated by 1,000 bootstrap replications, and curated in iTOL. The reference sequences retrieved from NCBI are highlighted in yellow in the phylogenetic tree.

16S rRNA gene sequencing results revealed that the isolates belonged to different taxonomic groups, as shown in Table 3. All examined isolates were classified into three phyla: *Pseudomonadota, Bacillota*, and *Actinomycetota* (Figure 2). Among these, 54.5% of the isolates belonged to *Pseudomonadota*, 40.9% to *Bacillota*, and 4.78% to *Actinomycetota*. At the genus level, *Pseudomonas* was predominant in the middle core (CB2), followed by the upper core (CB1) samples. *Bacillus* was observed in both the middle and upper core samples, while *Paenibacillus* and *Lysinibacillus* were prevalent in the bottom core (CB3) samples.

**Table 3:** Identified bacterial isolates based on 16S rRNA gene sequencing method.

**Ice core sample**	**Isolate code**	**Nearest phylogenetic species from GenBank database**	**Accession no**.	**Percent identity (in %)**
CB1	CB1.11	*Janthinobacterium* sp.	PV668848	100
	CB1.15	*Janthinobacterium* sp.	PV668849	100
	CB1.2	*Pseudomonas paraversuta*	PV668858	99.83
	CB1.18	*Bacillus thuringiensis*	PV668868	98.96
CB2	CB2.1	*Pseudomonas* sp.	PV668850	99.93
	CB2.8	*Pseudomonas gessardii*	PV668850	99.93
	CB2.13	*Paenarthrobacter ilicis*	PV668852	99.92
	CB2.21	*Pseudomonas gessardii*	PV668853	100
	CB2.5	*Pseudomonas pergaminensis*	PV668859	97.69
	CB2.19	*Bacillus cereus*	PV668860	99.92
	CB2.17	*Bacillus thuringiensis*	PV668861	99.92
	CB2.16	*Pseudomonas paraversuta*	PV668862	99.62
	CB2.20	*Bacillus thuringiensis*	PV668863	100
	CB2.22	*Bacillus thuringiensis*	PV668864	99.92
	CB2.25	*Pseudomonas pergaminensis*	PV668865	100
	CB3.7	*Lysinibacillus fusiformis*	PV668856	100
CB3	CB3.18	*Lysinibacillus fusiformis*	PV668854	100
	CB3.19	*Paenibacillus odorifer*	PV668857	100
	CB3.21	*Paenibacillus odorifer*	PV668855	100
	CB3.6	*Escherichia hominis*	PV668866	99.24
	CB3.4	*Escherichia ruysiae*	PV668867	98.48

#### 4.2.4 Antibiotic resistance profiles of glacier bacterial isolates

The results showed that most bacterial isolates exhibited resistance to Cefixime (CFM) (sensitive >19 mm, intermediate 16–18 mm, resistant <15 mm) and Methicillin (MET) (sensitive >15 mm, intermediate 10–13 mm, resistant <9 mm), followed by Ampicillin, Streptomycin, Nalidixic acid, Amoxicillin, and Erythromycin (Table 4). Gram-positive isolates from all three ice core samples showed resistance against β-lactam antibiotics. In brief, CB1.18 (upper core), CB3.5, and CB3.18 (bottom core) isolates showed resistance to Quinolone antibiotics; CB2.20 (middle core) and CB3.5 (bottom core) exhibited greater resistance to Aminoglycosides, and CB3.12 (bottom core) displayed resistance to multiple tested antibiotics. Among Gram-negative isolates, the upper core isolates (CB1) exhibited resistance to β-lactam, Aminoglycosides, and Quinolone antibiotics. Middle core isolates (CB2) showed resistance to multiple antibiotic groups, including β-lactams, Macrolides, Glycopeptides, and Chloramphenicol. Bottom core isolates displayed slight resistance to cephalosporins (β-lactam), while CB3.6 showed increased resistance toward Aminoglycosides. The susceptibility test profiles of all ice core isolates are summarized in Table 4. Overall, the majority of isolates from all three cores were susceptible to several tested antibiotics. Resistant isolates were predominant in the middle core (CB2), followed by the bottom core (CB3) and the upper core (CB1) (Supplementary Table 5).

**Table 4:** Antibiotic Susceptibility Pattern with corresponding MAR index value of individual bacterial isolates.

**Isolates**	**Resistant**	**Intermediate**	**Sensitive**	**MAR index**
CB1.1	NA, CIP, CFM	OF	AMC, GEN, TE, E, AMP, IMP, S, DO, VA, C, AZM, MET	0.18
CB1.2	NA, MET		AMC, GEN, TE, E, AMP, IMP, S, DO, VA, C, AZM, OF, CIP, CFM	0.12
CB1.7	CFM, S, MET		AMC, GEN, TE, E, AMP, IMP, DO, VA, C, AZM, OF, CIP, NA	0.18
CB1.6	CFM, S	NA	AMC, GEN, TE, E, AMP, IMP, DO, VA, C, AZM, OF, CIP, MET	0.12
CB1.18	NA, AMC, AMP, CFM		GEN, TE, E, IMP, DO, VA, C, AZM, OF, CIP, MET, S	0.25
CB1.19	AMC, AMP, IMP, MET		GEN, TE, E, DO, VA, C, AZM, OF, CIP, NA, CFM, S	0.25
CB1.11			AMC, GEN, TE, E, AMP, IMP, DO, VA, C, AZM, OF, CIP, NA, CFM, S, MET	0
CB1.15	CFM	NA	AMC, GEN, TE, E, AMP, IMP, DO, VA, C, AZM, OF, CIP, S, MET	0.06
CB1.23	AMC, AMP, CFM, S, MET		GEN, TE, E, IMP, DO, VA, C, AZM, OF, CIP, NA	0.31
CB1.13	AMC, AMP, S, MET		GEN, TE, E, IMP, DO, VA, C, AZM, OF, CIP, NA, CFM	0.25
**Mean** **±SD**				**0.17** **±0.09**
CB2.1	AMP, S, MET, VA	E	AMC, GEN, TE, IMP, DO, C, AZM, OF, CIP, NA, CFM	0.25
CB2.4	AMC, E, AMP, IMP, MET, NA, CFM, VA, C, AZM		GEN, TE, DO, OF, CIP, S	0.62
CB2.5	AMC, E, AMP, IMP, VA, C, AZM, CFM, S, MET	NA	GEN, TE, DO, OF, CIP	0.62
CB2.8	AMC, E, CFM, S, MET, AMP, VA	NA	GEN, TE, DO, C, AZM, OF, CIP, IMP	0.43
CB2.17	CFM		AMC, GEN, TE, E, AMP, IMP, DO, VA, C, AZM, OF, CIP, NA, S, MET	0.06
CB2.16	CFM	NA	AMC, GEN, TE, E, AMP, IMP, DO, VA, C, AZM, OF, CIP, S, MET	0.06
CB2.19	AMP	AMC	GEN, TE, E, IMP, DO, VA, C, AZM, OF, CIP, NA, CFM, S, MET	0.06
CB2.20	AMP, CFM, S	AMC	GEN, TE, E, IMP, DO, VA, C, AZM, OF, CIP, NA, MET	0.18
CB2.21	E, AMP, CFM, S, MET		AMC, GEN, TE, IMP, DO, VA, C, AZM, OF, CIP, NA	0.31
CB2.22	CFM, MET		AMC, GEN, TE, E, AMP, IMP, DO, VA, C, AZM, OF, CIP, NA, S	0.12
**Mean** **±SD**				**0.27** **±0.21**
CB3.4	CFM	NA	AMC, GEN, TE, E, AMP, IMP, DO, VA, C, AZM, OF, CIP, S, MET	0.06
CB3.5	NA, CFM, S, MET	CIP	AMC, GEN, TE, E, AMP, IMP, DO, VA, C, AZM, OF	0.25
CB3.6	CFM, S	NA	AMC, GEN, TE, E, AMP, IMP, DO, VA, C, AZM, OF, CIP, MET	0.12
CB3.7	CFM, MET		AMC, GEN, TE, E, AMP, IMP, DO, VA, C, AZM, OF, CIP, NA, S	0.12
CB3.12	AMC, GEN, TE, E, AMP, IMP, DO, VA, C, AZM, OF, CIP, NA, CFM, S, MET			1
CB3.13	MET		AMC, GEN, TE, E, AMP, IMP, DO, VA, C, AZM, OF, CIP, NA, CFM, S	0.06
CB3.15	CFM	NA	AMC, GEN, TE, E, AMP, IMP, DO, VA, C, AZM, OF, CIP, S, MET	0.06
CB3.18	NA, CFM		AMC, GEN, TE, E, AMP, IMP, DO, VA, C, AZM, OF, CIP, S, MET	0.12
CB3.19			AMC, GEN, TE, E, AMP, IMP, DO, VA, C, AZM, OF, CIP, NA, CFM, S, MET	0
CB3.21			AMC, GEN, TE, E, AMP, IMP, DO, VA, C, AZM, OF, CIP, NA, CFM, S, MET	0
**Mean** **±SD**				**0.17** **±0.29**

The tested antibiotics to which the isolates exhibited susceptible, intermediate and resistant profiles are shown in the table. The blank spaces in the table indicate that the respective antibiotics did not fall into that category for the given isolate. The isolates were tested against the following group of antibiotics: E, Erythromycin; CFM, Cefixime; TE, Tetracycline; C, Chloramphenicol; NA, Nalidixic acid; AMP, Ampicillin; IMP, Imipenem; AZM, Azithromycin; MET, Methicillin; CIP, Ciprofloxacin; VA, Vancomycin; S, Streptomycin; OF, Ofloxacin; GEN, Gentamycin; AMC, Amoxicillin; DO, Doxycycline hydrochloride. (Bauer 1996). ^*^(*n* = 10).

Evaluation of the multiple antibiotic resistance (MAR) index revealed heterogeneity in resistance patterns. Four isolates from the upper core (CB1.18, CB1.19, CB1.23, and CB1.13) had MAR indices greater than 0.2. From the middle core (CB2), five isolates (CB2.1, CB2.4, CB2.5, CB2.8, and CB2.21) exceeded this threshold, while only two isolates from the bottom core (CB3.5 and CB3.7) did so. The mean MAR index for CB1 was 0.17 ± 0.09, suggesting a relatively uniform resistance pattern among upper core isolates. For CB2, the mean MAR index was 0.27 ± 0.21, indicating moderate variability, and for CB3, the mean MAR index was 0.17 ± 0.29, reflecting high heterogeneity among bottom core isolates. The antibiotic susceptibility profiles along with corresponding MAR index values are presented in Table 4.

#### 4.2.5 Determination of minimum inhibitory concentration for antibiotics

The results revealed that most of the bacterial isolates exhibited higher MIC values for β-lactam antibiotics (penicillin, ampicillin, and amoxicillin; cephalosporin—cefixime) and aminoglycosides (streptomycin) (Table 5). Analysis of individual ice core isolates, as shown in Table 5, demonstrated that the middle core (CB2) isolates displayed MIC values greater than 16 μg L^−1^ for ampicillin, amoxicillin, cefixime, streptomycin, and methicillin. The MIC values for vancomycin fell within the intermediate range (4–8 μg L^−1^), whereas MIC values for erythromycin were <4 μg L^−1^ (susceptible range) for most middle core isolates. In the upper core (CB1), four isolates (CB1.18, CB1.19, CB1.23, and CB1.13) showed MIC values >16 μg L^−1^ for ampicillin and amoxicillin. The MIC values for methicillin and cefixime were <4 μg L^−1^ for most upper core isolates. In the bottom core (CB3), fewer isolates exhibited MIC values >16 μg L^−1^ for ampicillin, amoxicillin, cefixime, and methicillin.

**Table 5:** Minimum Inhibitory Concentration (μgL^−1^) assay of ice core samples.

**Samples**	**Antibiotics**	**Range of MIC value (**μ**gL**^**−1**^**)**			
		≤**2**	≤**4**	≤**8**	≥**16**
CB1	VA	2	4	3	1
	E	5	2	0	0
	S	0	3	3	4
	AMP	1	5	0	4
	AMC	1	5	0	4
	CFM	2	5	0	3
	MET	4	5	0	1
CB2	VA	0	4	4	2
	E	1	7	0	2
	S	3	3	0	4
	AMP	0	3	0	7
	AMC	0	3	2	5
	CFM	0	3	1	6
	MET	3	1	2	4
CB3	VA	2	6	2	0
	E	3	7	0	0
	S	1	7	0	2
	AMP	4	2	2	2
	AMC	4	3	2	1
	CFM	4	4	1	1
	MET	4	4	0	2

#### 4.2.6 Tolerance of glacier bacteria to heavy metals

The evaluation of MIC results revealed that most isolates from the three ice core samples exhibited higher tolerance to CuSO_4_, ZnCl_2_, and NiCl_2_, with MIC values ranging from 25 to 312.5 mM (Table 6). However, a low level of tolerance was observed to HgCl_2_, specifically in the bottom core (CB3), with an MIC value of <1 mM, as mercury is highly toxic even in lesser amounts. Core-wise analysis revealed that upper core (CB1) isolates showed the highest tolerance to ZnCl_2_, followed by middle core (CB2) and bottom core (CB3) isolates. In the case of CoCl_2_, most of the middle core (CB2) isolates showed higher tolerance, followed by the upper core (CB1) and bottom core (CB3) isolates, with MIC values ranging from 3.12 to 12.5 mM. The bottom core isolates displayed the capability to tolerate CuSO_4_ at higher concentrations (≤312.5 mM) than the upper and middle core isolates.

**Table 6:** Minimum inhibitory concentration (MIC) assay of heavy metals.

**Isolates**	**HgCl_2_ (mM)**	**NiCl_2_ (mM)**	**ZnCl_2_ (mM)**	**CuSO_4_ (mM)**	**CoCl_2_ (mM)**
CB1.1	0.78	50	50	25	6.25
CB1.2	0.39	125	50	25	6.25
CB1.6	2.5	50	50	25	12.5
CB1.7	2.5	50	50	25	12.5
CB1.18	0.39	12.5	50	12.5	12.5
CB1.19	0.39	12.5	50	12.5	3.125
CB1.11	0.78	12.5	50	12.5	6.25
CB1.15	0.78	50	50	12.5	6.25
CB1.13	0.156	12.5	25	6.25	12.5
CB1.23	0.156	12.5	50	12.5	12.5
CB2.8	0.78	25	3.12	25	12.5
CB2.1	0.78	25	50	25	6.25
CB2.21	0.78	25	50	25	12.5
CB2.4	0.195	25	50	50	12.5
CB2.16	1.25	12.5	25	6.25	12.5
CB2.20	0.078	12.5	25	12.5	12.5
CB2.5	0.39	12.5	50	25	12.5
CB2.17	0.078	12.5	25	6.25	12.5
CB2.22	0.78	12.5	50	12.5	12.5
CB2.19	0.78	12.5	50	6.25	12.5
CB3.4	0.019	25	50	312.5	12.5
CB3.6	0.156	12.5	50	12.5	12.5
CB3.7	0.0097	12.5	50	156	12.5
CB3.18	0.312	12.5	25	12.5	6.25
CB3.19	0.019	12.5	25	156	6.25
CB3.21	1.25	12.5	50	3.12	3.12
CB3.5	0.019	12.5	25	312.5	12.5
CB3.15	0.0097	50	12.5	156	6.25
CB3.13	0.078	50	50	156	6.25
CB3.12	0.078	12.5	12.5	312.5	3.12

### 4.3 Culture-independent

#### 4.3.1 16S rRNA gene (amplicon) sequencing

The outcome of high-throughput sequencing using the Illumina MiSeq platform yielded a total of ~18 million raw reads. In the output we obtained, 87,847 high-quality reads (non-chimeric, error-corrected reads) out of 316,664 processed reads and ASVs (final denoised unique sequences) from ice core samples of Kabru Glacier using the LotuS2 pipeline in the Galaxy platform (https://usegalaxy.eu/) (Özkurt et al., 2022). A total of 160 ASVs from the CB1 sample (upper core), 149 ASVs from the CB2 sample (middle core), and 185 ASVs from the CB3 sample (bottom core) were inferred after the high-quality reads were processed through DADA2 (implemented with default parameters in the LotuS2 pipeline). Therefore, the ASV counts represent the unique amplicon sequence variants detected after denoising. No amplification was observed in the extraction/PCR controls, indicating negligible background contamination throughout the workflow. The data for pre- and post-processing of amplicon reads, along with their corresponding SRA run accession numbers, are presented in Supplementary Table 6.

**Figure 3:**
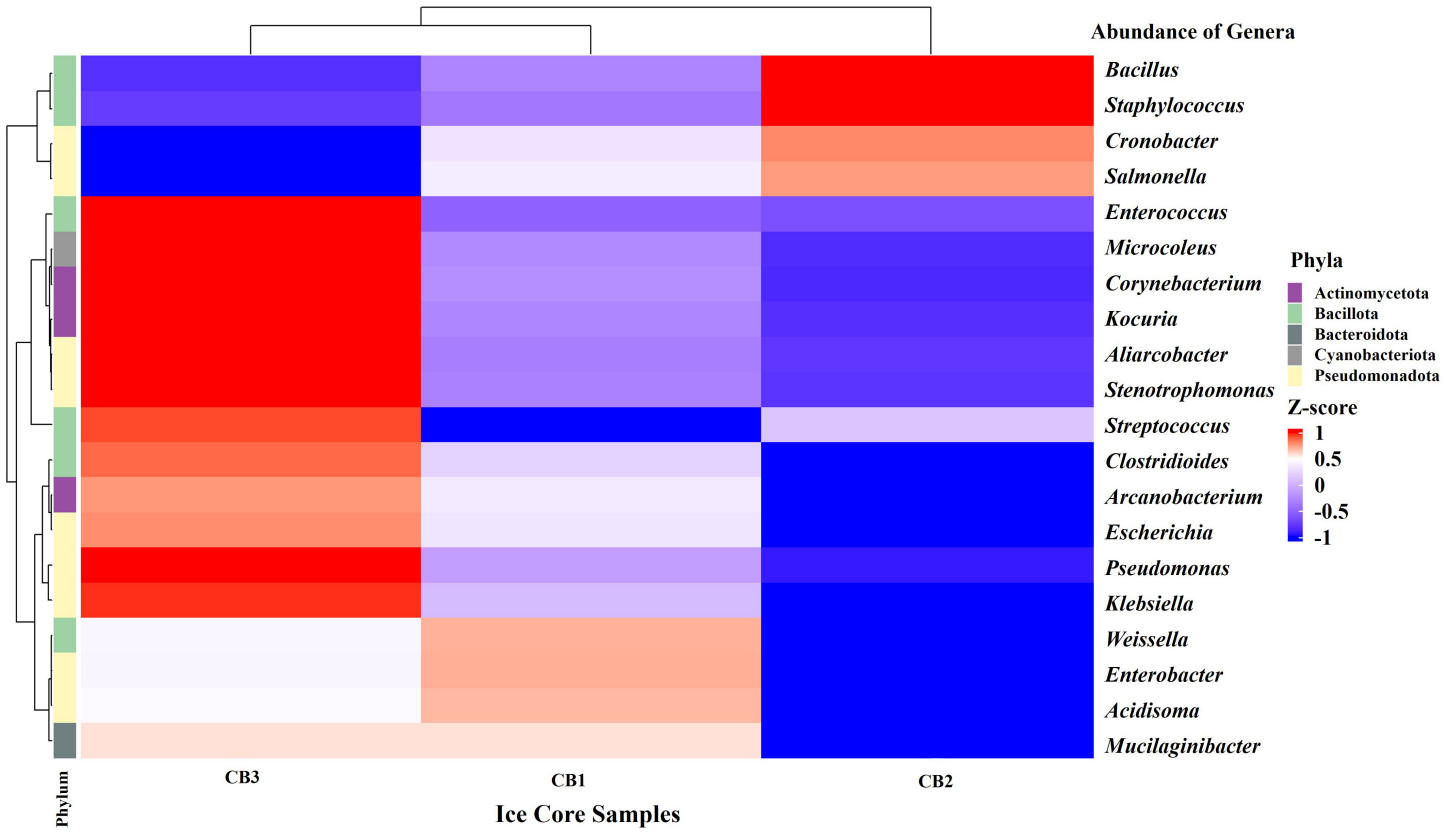
Heatmap of the top 20 abundant genera in the glacier ice core sample, with the depth of the color of cells in the matrix indicating the relative percentage of genera in each sample. Each row represents a genus, and the row annotation indicates its corresponding phylum (which is color-coded for clarity). The dendrogram, organized in rows and columns, results from a clustering calculation that depicts the dissimilarity in the relative abundance of genera across different glacier ice core samples.

The bacterial community in the Kabru Glacier ice core was predominantly composed of the phyla *Pseudomonadota* (52.1%), *Bacillota* (37.3%), *Bacteroidota* (6.3%), and *Actinomycetota* (1%). Phyla such as *Cyanobacteriota* and *Campylobacterota* were present at low relative abundance (<1%) and were limited in the ice core samples. The relative abundance of bacterial phyla is shown in Supplementary Figure 1. However, the distribution of genera in the glacier ice core sample was variable. A total of 36 genera were identified in the ice core samples, with genera such as *Cronobacter, Staphylococcus, Bacillus, Mucilaginibacter, Escherichia*, and *Pseudomonas* being predominant. Core-wise analysis revealed that *Cronobacter, Staphylococcus, Bacillus*, and *Enterococcus* were abundant in the middle core (CB2), followed by the upper core (CB1) and the bottom core (CB3), while *Escherichia* and *Pseudomonas* were prevalent in the bottom core. Elucidating the hierarchically clustered heatmap revealed that the bottom core (CB3) and upper core (CB1) of Kabru Glacier clustered together, indicating the relatedness of the bacterial composition at the genus level to some extent (Figure 3). The relative abundance values of the identified genera are presented in Supplementary Table 7.

#### 4.3.2 Assessment of alpha diversity

**Figure 4:**
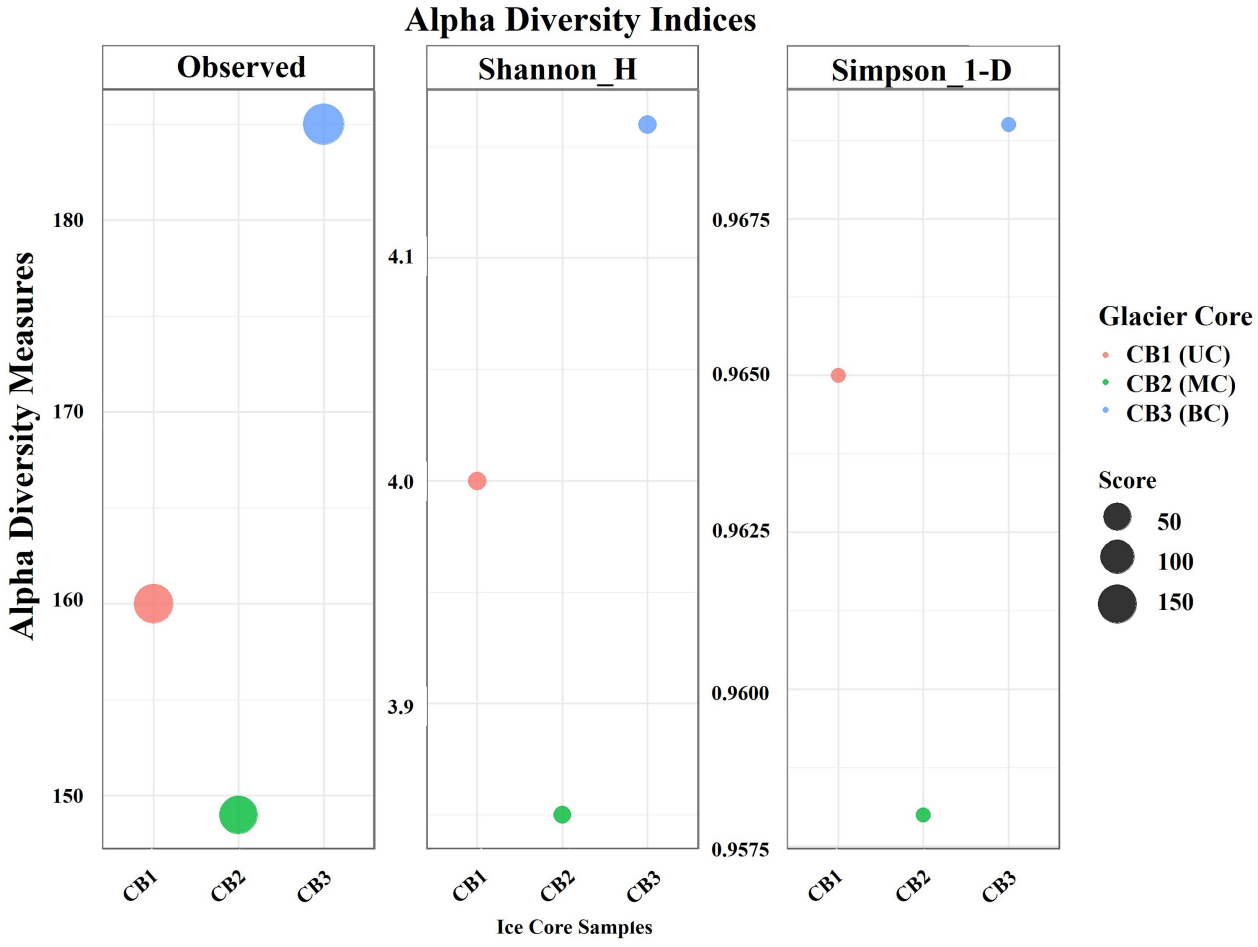
Alpha diversity of species abundance in the ice core sample.

The assessment of alpha diversity indices, such as Shannon-H and Simpson-1-D, revealed the bacterial community's species richness and evenness. The calculated alpha diversity indices showed a slight difference among the ice core samples studied. The bottom core (CB3) appears to exhibit higher microbial diversity, with an observed richness of 185 ASVs, a Shannon-H index of 4.16, and a Simpson 1-D index of 0.969, suggesting greater richness and evenness compared to the upper core (CB1) and the middle core (CB2). The alpha diversity is represented in Figure 4, and the calculated diversity indices for the three samples are detailed in Supplementary Table 8.

#### 4.3.3 Correlation analysis of culture-dependent, culture-independent diversity metrics in relation to geochemical elements

**Figure 5:**
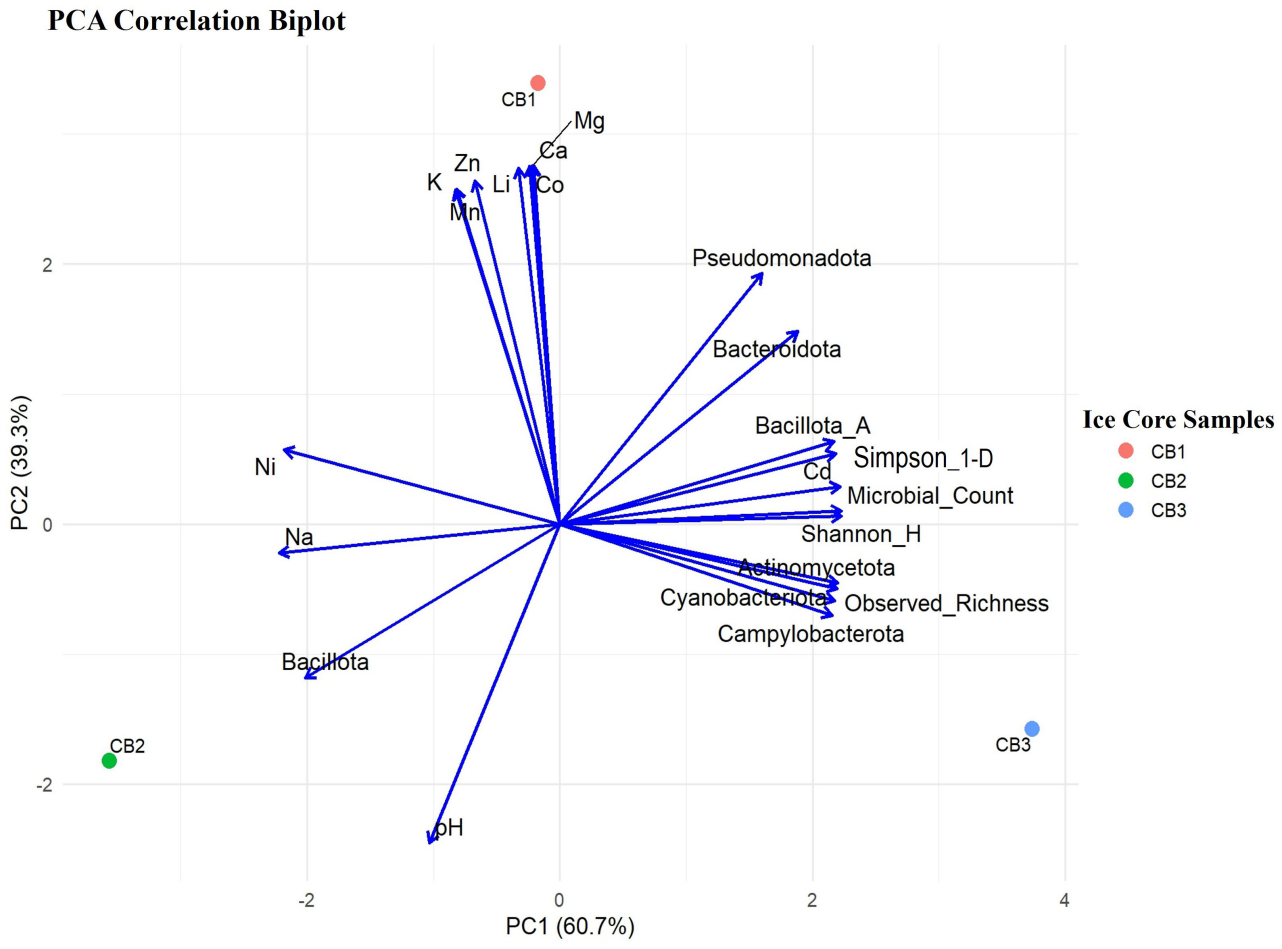
PCA plot showing major bacterial phyla, alpha diversity (culture-independent), and physicochemical parameters. Colored dots represent ice core samples (CB1, Upper; CB2, Middle; CB3, Bottom); arrows indicate variables, with the lengths of the arrows showing their contributions.

PCA was used as a descriptive visualization tool to examine the potential association among microbial and geochemical variables across the three ice core samples. The PCA results revealed that PC1 (60.7%) explained the major variation, followed by PC2 (39.3%), representing the secondary variation in the Kabru ice core sample (Figure 5). The ordination plot suggested that bacterial CFU counts, alpha diversity metrics, and abundant phyla (*Pseudomonadota* and *Bacteroidota*), along with less abundant phyla (*Actinomycetota, Campylobacterota*, and *Cyanobacteriota*), may co-vary and appear associated with the bottom ice core sample (CB3). In contrast, major elements (K, Mg, Ca, Li, and Mn) and certain heavy metals (Zn and Co) were aligned closer to the upper ice core (CB1), which could reflect the influence of atmospheric deposition and geochemical accumulation. However, to interpret these patterns further, individual validation via stratigraphy, dust load, and chronological data would be required. The pH appeared inversely aligned with the geochemical variables associated with the bottom ice core. However, pH was in visual proximity to the abundant phylum *Bacillota* and Na+ ion in the middle ice core region (CB2).

## 5 Discussion

The current study focuses on bacterial diversity in the ice core sample from Kabru Glacier, utilizing both culture-dependent and independent methods, as well as examining the antibiotic resistance and metal-tolerant properties of the bacterial isolates. In the culture-dependent study, a total of 75 bacterial species were retrieved from the different depths of the ice core sample (upper, middle, and bottom core). The bacterial load was determined from each ice core section based on the enrichment method, revealing that the highest bacterial count was recorded from the bottom core (CB3) at 7.0 × 10^8^ CFU mL^−1^, followed by the upper core (CB1) at 3.5 × 10^8^ CFU mL^−1^, and the middle core (CB2) at 5.9 × 10^6^ CFU mL^−1^. Bacterial counts in the ice core sections of various non-polar glaciers have highlighted a higher bacterial load than in polar glaciers; for example, the ice core from Yuzhufeng Glacier on the Tibetan Plateau recovered between ~ 7 and 6,667 CFU mL^−1^ (Shen et al., 2018), while from the Puruogangri Glacier, CFU ranged from 10^4^ to 10^5^ cell mL^−1^ (Zhang X. F. et al., 2008), and from the East Rongbuk Glacier, the culturable bacterial concentration varied from 0 to 1,720 CFU mL^−1^ along the depth (Shen et al., 2012). In our previous study, the culturable bacteria recovered from the Changme Khang Glacier and Changme Khangpu Glacier located in the Sikkim Himalaya were 1.5 × 10^4^ cells mL^−1^ and 1.5 × 10^5^ cells mL^−1^, respectively (Sherpa et al., 2018a). The accounts of bacterial load in polar glaciers are relatively lower, suggesting that the geographic location of these glaciers, as extreme polar counterparts, and the influence of atmospheric circulation may play a pivotal role in variations of culturable bacterial load. Moreover, the concentration of bacterial cells in glacial ice is correlated with the presence of dust and climate change (Zhang S. et al., 2008; Tamang et al., 2023).

The recovery of culturable bacteria is notably higher in non-polar glaciers due to their proximity to regions that are sources of aerosols carrying high microbial biomass (Shen et al., 2018). Moreover, the rich biodiversity in the adjoining region influences the diversity of microorganisms that accumulate in non-polar and Himalayan glaciers (Hodson et al., 2008; Christner et al., 2000). The account of microbial diversity from non-polar glaciers has revealed *Pseudomonadota* (formerly Proteobacteria), *Bacteroidota* (formerly Bacteroidetes), *Cyanobacteriota* (formerly Cyanobacteria), *Bacillota* (formerly Firmicutes), *Actinomycetota* (formerly Actinobacteria), and *Verrucomicrobiota* (formerly Verrucomicrobia) as the dominant bacterial phyla, as environmental factors largely influence their survival in the cold environment (Sharma et al., 2020). Sherpa et al. reported the abundance of *Pseudomonadota* (formerly Proteobacteria) (56%), followed by *Bacillota* (formerly Firmicutes) (16%) and *Actinomycetota* (formerly Actinobacteria) (12%) in the glacier moraine soil of the Changme Khangpu Glacier in the Sikkim Himalaya (Sherpa et al., 2020). Therefore, the Himalayan glaciers serve as biodiversity hotspots for cold-adapted microorganisms. In this study, variation in bacterial load was detected at different depths of the glacier. The highest number of bacterial counts was recorded from the bottom core (CB3) of the Kabru Glacier. Similarly, bacterial colonies from the ice core sample of Yuzhufeng Glacier on the Tibetan Plateau exhibited an increasing trend with depth (Shen et al., 2018). However, in this study, the middle core (CB2) showed a lower bacterial load. The variation in the recovered culturable bacterial colonies across different ice core sections indicates ecological zonation within the glacier, possibly due to the availability of organic and inorganic matter at varying depths within the ice core. Additionally, the recovery of bacteria in different culture media highlights the varying nutrient requirements of these bacteria (Stewart, 2012; D'Andrilli and McConnell, 2021; Liu et al., 2019; Shen et al., 2018). The recovery of microorganisms from different depths of the ice core sample of the glacier depends on the availability of nutrients, the influence of climate, and the regional environment (Xiang et al., 2005).

The species diversity identified at all three depths of the ice core based on 16S rRNA gene sequences showed consistent results with studies from various glaciers (Liu et al., 2019; Shen et al., 2012, 2018; Sherpa et al., 2018a; Xiang et al., 2005; Zhang X. F. et al., 2008). However, the distribution of major phyla (in this study, *Pseudomonadota, Bacillota*, and *Actinomycetota*) varied in ice core samples from geographically separated glaciers, as well as along the depth of the ice core. The deposition of diverse taxonomic groups of bacteria in the glacier ice core reflects the influence of the Indian monsoon in summer and the westerlies in winter, which transport microorganisms from surrounding habitats and deposit them in the glacier through precipitation (Shen et al., 2012; Zhang et al., 2007). The prevalence of different bacterial taxa along the depth of the ice core suggests their involvement in competition for limited nutrients and water content, indicating the survival strategies of these microorganisms to cope with the extreme environment (Xiang et al., 2005). In this study, pigment-producing bacterial isolates were primarily recovered from the middle core (CB2), and these isolates exhibited sequence similarity with the genus *Pseudomonas* in GenBank. Likewise, (Shen et al. 2012) reported the recovery of pigment-producing bacteria from the middle ice core sample of the East Rongbuk Glacier on Mt. Everest. Although two of the upper core isolates showed sequence similarity with *Janthinobacterium* sp., which are psychrotolerant bacteria commonly found in glacier environments, including alpine glacier cryoconite and Antarctic proglacial lakes (Kim et al., 2012; Koo et al., 2016), this organism produces a purple-violet pigment known as violacein, a key adaptive feature in harsh environments (Mojib et al., 2013; Wu et al., 2021). In our study, the probable reason for the accumulation of more Gram-negative pigment-producing bacterial isolates in the middle core may be their ability to recuperate and possibly protect themselves from harmful UV radiation due to the stable and more hospitable environment in the deeper section. Therefore, microbes inhabiting the middle core, which is a static environment, potentially utilize organic matter efficiently, subsequently enhancing their recovery and aiding in the expression of adaptive traits (pigment production). However, in the absence of depth-resolved UV flux data, this plausible adaptation remains a working hypothesis. In contrast, the upper core is under the influence of fluctuating UV and temperature, which may force the microorganisms into deep physiological dormancy as a survival strategy. Furthermore, we can speculate that in the bottom core, there is a depletion of nutrients and energy, resulting in a decrease in bacterial diversity along with the loss of adaptive traits (Shen et al., 2012, 2018). Thus, the difference in the distribution of the bacterial community in different sections of the ice core may reflect the bacterial response to climate change and their deposition during different time periods (Shen et al., 2012). Additionally, it directs our understanding toward the structure of dust and aerosols, which vary in each ice core and are potential carriers of microorganisms in the glacier.

The culture-independent study based on 16S rRNA gene (Amplicon) sequencing was employed to analyze the bacterial diversity in the Kabru glacier ice core samples. The results of the taxonomic classification revealed the predominance of *Pseudomonadota*, followed by *Bacillota, Bacteroidota*, and *Actinomycetota*. Although the mean percentage of the major phyla differed marginally, the relative abundance of individual bacterial phyla varied in each ice core sample, as mentioned earlier in our results. In our previous study, we identified similar major bacterial phyla in glacier samples from Changme Khang and Changme Khangpu Glacier, North Sikkim, India (Sherpa et al., 2021). The prevalence of *Pseudomonadota* (formerly Proteobacteria), *Bacillota* (formerly Firmicutes), *Bacteroidota* (formerly Bacteroidetes), and *Actinomycetota* (formerly Actinobacteria) in the glacier ice core samples has been reported in studies from the polar region, Antarctic (Shivaji et al., 2013) and Arctic counterparts (Miteva et al., 2004, 2015), as well as in non-polar regions such as the Himalaya and Tibetan Plateau (Sherpa et al., 2018a; Shen et al., 2018, 2012). The distribution of major phyla in this study varied along the vertical section of the ice core samples obtained from the glaciers of the Sikkim Himalaya. This suggests that environmental factors, such as temperature, precipitation, and most importantly, global atmospheric circulation, may influence the bacterial diversity of the region (Zhang et al., 2001; Yao et al., 2008). In addition, the heterogeneity in bacterial genera across different layers of the ice core may reflect the influence of aeolian processes on the deposition of microbial communities under past climatic conditions, although this interpretation remains speculative due to the lack of individual validation through stratigraphy and atmospheric data. Moreover, a key limitation of this study is the reliance on a single ice core sample, which restricts biological replication. Therefore, to partially address this, we carried out culture assays in triplicate and pooled technical PCR replicates to enhance reproducibility and reduce bias. Future studies should incorporate replicate ice cores to improve statistical robustness and strengthen ecological inference. The alpha diversity assessment in the present study suggests that the bacterial diversity in the bottom ice core of the Kabru Glacier appears to be higher in terms of species richness and evenness. This observation regarding alpha diversity was consistent with the total bacterial count, suggesting that the bottom core may support a more diverse and abundant bacterial community, even in a nutrient-poor region (Segawa et al., 2017). Although a minor difference in the bacterial community was observed within the samples, a substantial difference in the bacterial composition between the three ice core samples was noted. These differences are probably driven by various factors, including aeolian processes and environmental parameters such as pH, trace elements, and other ions, which collectively shape the bacterial community structure in the glacier ice. These findings were corroborated by Principal Component Analysis, which highlighted the possible associations among different variables that ultimately support microbial life in extreme environments. However, the associations made are exploratory and qualitatively interpreted, as the sample size was limited in this study.

Assessing abiotic factors in the glacier is necessary because climate change can significantly impact these elements, which in turn affect the glacier's dynamics, structure, water resources, and regional ecosystem. The complex aerosol composition in the Himalayas has both natural and anthropogenic origins, creating heterogeneity in the deposition of elements at different geographic locations on glaciers (Barrie, 1985). A disparity in the distribution of elements was observed in the three ice core samples in this research. The concentrations of Na, Mg, K, Ca, and Mn (in ppb) were found to be higher in the upper core (CB1), followed by the middle core (CB2), and the bottom core (CB3). The higher concentration of TDS (total dissolved solids) recorded from the upper core of the glacier suggests the influence of bedrock weathering from the surrounding area and the effect of maritime aerosols (Shen et al., 2018). The presence of heavy metals, viz. Cu, Ni, Zn, and Co, in the three ice core samples was also noted. Likewise, the occurrence of heavy metals in glacier ice cores has been documented from both polar and non-polar glaciers (Eyrikh et al., 2017; Gabrielli and Vallelonga, 2015; Jiao et al., 2021; Kumar et al., 2025; Thamban and Antony, 2023; Ferrari et al., 2001). These contaminants found in glacier ice cores may originate from anthropogenic activities, such as mining and industrial inputs, and are transported to remote locations through the atmosphere (Gabrielli and Vallelonga, 2015).

The growth profile study of the 30 selected isolates revealed that the optimum growth temperature was in the range of 15 °C to 20 °C. However, most of the isolates were able to grow at 37 °C; the isolates CB3.7, CB3.18 (bottom core), and CB1.18 (upper core) exhibited optimum growth at this temperature. The ability to grow in the range of 10 °C to 37 °C suggests that these isolates are affiliated with psychrotolerant species/genera, excluding those associated with the genus *Lysinibacillus* (which has an optimum temperature of 37 °C). The presence of mesophilic bacteria in the ice core region from various cryospheric environments has been reported, specifically from glaciers in the polar region, the Tibetan Plateau, and the Himalayan range (Liu et al., 2019; Rafiq et al., 2019; Shen et al., 2012; Shivaji et al., 2013). The mesophiles in the glacier indicate a warmer climate in the past and specific conditions that may favor the survival of these bacterial isolates. Additionally, most isolates showed growth at 0.5%−5% NaCl concentration (with optimum growth at 1% NaCl), and the optimum pH for the growth of the majority of ice core isolates was between pH 5 and pH 9. Extremophiles exhibit remarkable adaptability to extreme environmental conditions, with wide pH tolerance being a significant characteristic (Giovanella et al., 2020; Dhakar and Pandey, 2016). The hypothetical basis for their adaptation may lie in proteome adaptability (which involves changes in the composition/expression of proteins due to shifts in ecological conditions) and the presence of specific genetic compositions (Dhakar and Pandey, 2016). Therefore, the observed pH tolerance of isolates represents a general phenotypic characteristic but may also have ecological relevance. These results suggest that the isolates not only possess cold adaptation but can also withstand moderate salinity and variable pH conditions, which may be ecologically significant given the fluctuations in glacier meltwater chemistry primarily caused by mineral dissolution, atmospheric deposition, and microbial metabolism (Varliero et al., 2023).

Moreover, (Wilson et al. 2012) have mentioned the phenomenon of cross-tolerance, which occurs when bacteria are subjected to osmotic stress, driving the production of cold shock proteins (CSPs), an antifreeze protein that ultimately helps the organism survive freezing temperatures. This observation was reported from hyperosmotic environments. Hence, we can hypothesize that most of our bacterial isolates from the upper and middle core, which were able to grow at 5% NaCl concentration, are experiencing this cross-tolerance phenomenon.

Another concerning factor in the glacier environment is the prevalence of antibiotic resistance genes and bacteria. Antibiotic-resistant bacteria have been reported in ice core samples retrieved from various geographically located glaciers (Nawaz et al., 2023; Rafiq et al., 2017, 2019; Segawa et al., 2013; Sherpa et al., 2020). In pristine environments, such as glaciers, the dissemination of antibiotic resistance genes has been speculated to occur through global air circulation and the excrement of migratory birds (Segawa et al., 2013; Sherpa et al., 2020; Sjölund et al., 2008). This study highlighted the presence of higher antibiotic-resistant bacterial isolates in the middle core (CB2) of Kabru Glacier, followed by the upper core (CB1) and the bottom core (CB3). Furthermore, the determination of the multiple antibiotic resistance (MAR) index of the Kabru Glacier isolates revealed that the middle core (CB2) isolates displayed resistance to multiple antibiotics, with comparatively lesser variability in the resistance pattern among the isolates (0.27 ± 0.21). This suggests that most of the middle core isolates acquire specific resistance mechanisms (target modification, efflux pump), which might be induced by natural stressors (heavy metals, competition among microbes, lower temperature), leading to uniform resistance among the isolates (Brepoels et al., 2025; Frost et al., 2018). Similar to our findings, multiple antibiotic resistance (MAR index > 0.5) has been reported from the Changme Khang and Changme Khangpu Glaciers, located in the Sikkim Himalayas, and from the Siachen Glacier, located in the Karakoram range of the Himalayas (Sherpa et al., 2020; Rafiq et al., 2017). This result indicates that multiple antibiotic-resistant bacteria can be recovered even from pristine environments, such as glaciers, where there is no anthropogenic influence. Hence, it demands an elaborate investigation to gain insight into the mechanisms of the spread of antibiotic resistance genes in these cold-adapted microbial communities. The identification of isolates through 16S rRNA gene sequencing and observations of the Gram staining nature revealed that Gram-negative bacterial isolates were predominantly present in the middle core (CB2). Gram-negative bacteria possess an outer membrane (absent in Gram-positive bacteria), which provides extra protection to the bacteria. The lipid bilayer filters out molecules based on their size and shape, acting as a selective barrier that confers an advantage on these bacteria and enables them to resist various hydrophobic antibiotics. Moreover, these Gram-negative bacteria can acquire various resistance mechanisms through the modification of efflux pumps and porin proteins, enabling them to survive under antimicrobial stress. Another important point, in addition to cellular and molecular adaptation in response to antibiotics in the environment, is that Gram-negative bacteria, such as *Escherichia coli* and *Pseudomonas aeruginosa*, are pathogens that primarily transfer antibiotic resistance genes through mobile genetic elements (MGEs) (Gauba and Rahman, 2023). As Gram-negative organisms require minimal nutrients and possess various resistance mechanisms, this may explain their higher resistance to most antibiotics (Yitayeh et al., 2021; Oliveira and Reygaert, 2023). Therefore, the presence of antibiotic-resistant Gram-negative isolates in the glacier highlights the need for a thorough investigation of these environments. In comparison, Gram-positive isolates were prevalent in the bottom core (CB3). The 16S rRNA gene sequence identified isolates as spore formers, rod-shaped, which showed sequence similarity with species belonging to *Bacillota* in the GenBank database. The survival of spore formers in the deeper regions of the ice core suggests their resilience to a harsh environment, as evidenced by the formation of dormant spores. Moreover, these Gram-positive bacterial isolates, which exhibit resistance to the tested antibiotics, raise greater concern for global health because they can acquire resistance to multiple antibiotics, similar to some clinically important bacterial strains (Jubeh et al., 2020).

The assessment of the metal tolerance ability of the glacier isolates is crucial, as these isolates can be advantageous for bioremediation purposes in cold environments (Hoover and Pikuta, 2010). The metal-tolerating bacterial isolates have been reported from various habitats of the Laohugou Glacier (LHG), China (Ali et al., 2019), from the Siachen Glacier, Pakistan (Rafiq et al., 2017), and from the glaciers of North Sikkim, India (Changme Khang and Changme Khangpu Glacier) (Sherpa et al., 2020). In our study, bacterial isolates from three glacier ice core samples were evaluated for their heavy metal tolerance capabilities, and it was observed that the isolates were tolerant to CuSO_4_, ZnCl_2_, and NiCl_2_. This finding was consistent with our previous study from the Changme Khang and Changme Khangpu Glacier, Sikkim Himalaya (Sherpa et al., 2020). Additionally, the concentration of metal ions in the ice core samples, detected through ICP-MS, was significantly higher in the upper core. Likewise, heavy metal tolerance was observed mostly in the upper ice core, except for CuSO_4_, which was higher in the bottom core. Since most of the bottom and upper core isolates were Gram-positive, it can be inferred that Gram-positive bacteria with a thicker peptidoglycan layer have a greater capacity for metal binding, which allows for higher accumulation of metals in their cell walls (Cufaoglu et al., 2022).

This suggests that the heavy metal-tolerating bacterial isolates in the glacier might be actively involved in the biogeochemical cycle and highlights their potential in biotechnological applications. Furthermore, the co-selection theory suggests that the presence of heavy metals in the environment leads to the co-selection of metal and antibiotic resistance in microbes via mechanisms like co-resistance (metal-tolerating genes and antibiotic resistance genes are present on the same mobile genetic elements) and cross-resistance (sharing common mechanisms to expel the toxic elements, such as efflux pumps) (Sherpa et al., 2020; Di Cesare et al., 2016; Murray et al., 2024). Hence, the occurrence of antibiotic resistance in the glacier environment may be driven by heavy metal leaching from glacier-derived geological formations (Sherpa et al., 2020; Romaniuk et al., 2018). In essence, the prevalence of both metal resistance and antibiotic resistance, even in pristine glacier environments, highlights the intricate ecological interactions between these adaptive factors.

## 6 Conclusions

The present study provides the first comprehensive investigation of bacterial diversity from glacier ice cores in the Sikkim Himalayan region, marking a pioneering effort in understanding the bacterial ecology of Kabru Glacier. Both culture-dependent and culture-independent approaches were employed to unravel the bacterial community structure and functional potential. Physicochemical analyses revealed slightly acidic pH across all ice cores with variable elemental concentrations, particularly elevated levels of Na, Mg, K, Ca, Mn, Li, and Zn in the upper core. Culture-dependent analysis revealed an elevated bacterial load in the bottom core, with Gram-positive isolates predominating across all core sections, although Gram-negative bacteria were more prevalent in the middle core. Although this observation in a culture-dependent study demands further comprehensive investigation, the observed CFU counts in this study represent the viable bacterial biomass that proliferated under enrichment conditions. Hence, the bacterial load may not reflect the absolute microbial abundance present in the *in situ* glacier ice environment. However, morphological, biochemical, and enzymatic characterisation highlighted considerable metabolic versatility among the isolates, while phylogenetic analysis placed them within *Pseudomonadota, Bacillota*, and *Actinomycetota*. Compared to culture-dependent approaches, amplicon-based sequencing provided deeper insights into the *in situ* glacier bacterial diversity, confirming *Pseudomonadota* and *Bacillota* as dominant phyla alongside *Bacteroidota* and *Actinomycetota*. A distinct contrast emerged between the two approaches: cultivation methods recovered genera such as *Pseudomonas, Bacillus, Paenibacillus*, and *Micrococcus*, while sequencing revealed ecologically significant but uncultured or fastidious taxa, including *Polaromonas, Janthinobacterium, Flavobacterium*, and *Sphingomonas*. This difference underscored the complementary value of both strategies and highlighted the presence of diverse bacterial lineages adapted to glacial environments but not easily retrievable under laboratory conditions. Antibiotic susceptibility and MIC assays revealed multidrug resistance in several isolates, particularly in the middle core, while heavy metal assays demonstrated high tolerance to Zn, Cu, and Ni, with Gram-positive bacteria showing greater resistance. Diversity analyses suggest greater richness and evenness in the bottom core, and PCA showed potential associations between bacterial diversity, elemental composition, and physicochemical parameters. A key limitation of this study was the absence of total organic carbon (TOC), dissolved organic carbon (DOC), and inorganic carbon profiling, which could have strengthened the links between bacterial diversity, carbon cycling, and paleoclimatic interpretations. Future investigations that integrate these parameters with metagenomics will enable a more comprehensive understanding of glacial biogeochemistry. Importantly, climate-driven glacial retreat may lead to the release of ancient bacterial communities into downstream ecosystems. Rising temperatures may alter their composition, potentially affecting nutrient cycling, biogeographic patterns, and the dissemination of antibiotic resistance genes. As the first ice-core-based bacterial study in the Sikkim Himalaya, these findings not only expand baseline knowledge but also highlight the ecological and biotechnological significance of Himalayan glaciers in the era of rapid climate change.

## Data Availability

The assembled amplicon sequencing data have been deposited in the NCBI Sequence Read Archive (SRA) under accession number PRJNA1109674. The 16S rRNA gene sequences of representative isolates from Kabru Glacier ice cores have been submitted to NCBI under accession numbers PV668848-PV668868.
