# Supplementary material for: Exploring the bacterial diversity and its antibiotic resistance in Kabru Glacier ice cores, Sikkim Himalaya
Source: Front Microbiol. 2026 Jan 28;16:1672943. doi: 10.3389/fmicb.2025.1672943 (PMC12893349; doi:10.3389/fmicb.2025.1672943)
Supplement: Supplementary file 1 [file Data_Sheet_1.ZIP › Supplementary folder/STNewRev Supplementary Table.docx]

**Supplementary Table 1:** Morphological characteristics and biochemical properties of the bacterial isolates.

| **ISOLATES** | **MORPHOLOGICAL CHARACTERISTICS** | **BACTERIAL SHAPE** | **GRAM STAIN** | **INDOLE TEST** | **METHYL-RED TEST** | **VOGES-PROSKAUER**  **TEST** | **CITRATE TEST** | **NITRATE REDUCTION TEST** |
| --- | --- | --- | --- | --- | --- | --- | --- | --- |
| **CB1.1** | Entire, white, convex, shiny | Rod-shaped | −ve | + | + | + | + | - |
| **CB1.2** | Entire, white, convex, shiny | Rod-shaped | −ve | - | + | - | + | - |
| **CB1.3** | Entire, white, convex, shiny | Rod-shaped | −ve | + | - | - | - | - |
| **CB1.4** | Entire, white, convex, shiny | Rod-shaped | +ve | + | - | - | - | - |
| **CB1.5** | Undulate, white, pulvinate, slimy | Rod-shaped | +ve | - | - | - | - | - |
| **CB1.6** | Entire, white, convex, shiny | Rod-shaped | −ve | - | - | + | + | + |
| **CB1.7** | Entire, creamy, convex, shiny | Rod-shaped | −ve | - | - | + | + | + |
| **CB1.8** | Entire, creamy, convex, shiny | Rod-shaped | +ve | - | - | - | + | - |
| **CB1.9** | Entire, white, convex, shiny | Rod-shaped | +ve | - | - | - | - | - |
| **CB1.10** | Entire, white, convex, shiny | Rod-shaped | +ve | - | - | - | - | - |
| **CB1.11** | Entire, white, convex, shiny | Rod-shaped | −ve | + | + | - | - | - |
| **CB1.12** | Entire, white, convex, shiny | Rod-shaped | +ve | - | - | - | - | - |
| **CB1.13** | Entire, white, convex, shiny | Rod-shaped | −ve | + | + | - | - | + |
| **CB1.14** | Entire, white, convex, shiny | Rod-shaped | −ve | - | - | - | + | - |
| **CB1.15** | Entire, white, convex, shiny | Rod-shaped | −ve | + | + | - | + | - |
| **CB1.16** | Curled, white, convex, shiny | Rod-shaped | −ve | - | - | - | - | - |
| **CB1.17** | Curled, creamy, flat, smooth | Rod-shaped | +ve | - | - | - | - | - |
| **CB1. 18** | Undulate, creamy, flat, dull | Rod-shaped | +ve | - | - | + | - | + |
| **CB1.19** | Undulate, creamy, flat, dull | Rod-shaped | +ve | - | - | + | - | + |
| **CB1.20** | Undulate, creamy, flat, dull | Rod-shaped | +ve | - | - | - | - | - |
| **CB1.21** | Curled, creamy, flat, dull | Rod-shaped | +ve | - | - | - | - | - |
| **CB1.22** | Curled, creamy, flat, dull | Rod-shaped | +ve | - | - | - | - | - |
| **CB1.23** | Curled, creamy, flat, dull | Rod-shaped | +ve | - | + | - | - | + |
| **CB1.24** | Curled, creamy, flat, dull | Rod-shaped | +ve | - | - | - | - | - |
| **CB1.25** | Even, white, convex, shiny | Rod-shaped | +ve | + | - | - | - | - |
| **CB2.1** | Curled, creamy, flat, shiny | Rod-shaped | −ve | + | - | - | + | + |
| **CB2.2** | Undulate, creamy, flat, shiny | Rod-shaped | +ve | - | - | - | - | - |
| **CB2.3** | Curled, creamy, flat, shiny | Rod-shaped | −ve | + | - | - | - | - |
| **CB2.4** | Curled, creamy, flat, smooth, dull | Rod-shaped | −ve | - | + | - | + | + |
| **CB2.5** | Undulate, white, flat, smooth, dull | Rod-shaped | −ve | - | + | - | + | + |
| **CB2.6** | Even, white, pulvinate, shiny | Rod-shaped | +ve | - | - | - | - | - |
| **CB2.7** | Curled, white, pulvinate, shiny | Rod-shaped | +ve | + | - | - | - | - |
| **CB2.8** | Curled, white, pulvinate, shiny | Rod-shaped | −ve | - | - | - | + | + |
| **CB2.9** | Even, white, pulvinate, shiny | Cocci-shaped | −ve | - | - | - | - | - |
| **CB2.10** | Even, white, pulvinate, shiny | Cocci-shaped | −ve | - | - | - | - | - |
| **CB2.11** | Even, white, pulvinate, shiny | Cocci-shaped | +ve | - | - | - | - | - |
| **CB2.12** | Even, white, pulvinate, shiny | Cocci-shaped | −ve | - | - | - | - | - |
| **CB2.13** | Even, white, pulvinate, shiny | Cocci-shaped | +ve | - | - | - | + | + |
| **CB2.14** | Even, white, pulvinate, shiny | Cocci-shaped | +ve | - | - | - | - | + |
| **CB2.15** | Even, white, pulvinate, shiny | Cocci-shaped | −ve | - | - | - | - | + |
| **CB2.16** | Even, white, pulvinate, shiny | Cocci-shaped | −ve | - | - | - | + | + |
| **CB2.17** | Curled, creamy, flat, smooth, dull | Rod-shaped | +ve | - | - | + | + | + |
| **CB2.18** | Curled, creamy, flat, smooth, dull | Rod-shaped | +ve | - | + | - | - | - |
| **CB2.19** | Curled, creamy, flat, smooth, dull | Rod-shaped | +ve | - | - | + | + | + |
| **CB2.20** | Curled, creamy, flat, smooth, dull | Rod-shaped | +ve | - | + | + | + | - |
| **CB2.21** | Curled, creamy, convex, shiny | Rod-shaped | −ve | - | - | - | + | + |
| **CB2.22** | Curled, creamy, flat, dull | Rod-shaped | +ve | - | + | + | + | + |
| **CB2.23** | Curled, white, convex, shiny | Rod-shaped | −ve | - | - | - | - | - |
| **CB2.24** | Curled, creamy, flat, dull | Rod-shaped | −ve | - | - | - | - | - |
| **CB2.25** | Even, creamy, convex, shiny | Rod-shaped | −ve | - | - | - | + | + |
| **CB3.1** | Undulate, creamy, convex, shiny | Cocci-shaped | +ve | + | + | - | - | + |
| **CB3.2** | Undulate, creamy, convex, shiny | Cocci-shaped | +ve | + | - | - | - | - |
| **CB3.3** | Entire, creamy, convex, shiny | Cocci-shaped | −ve | + | + | - | - | + |
| **CB3.4** | Entire, creamy, convex, shiny | Rod-shaped | −ve | + | + | - | - | + |
| **CB3.5** | Undulate, creamy, convex, shiny | Rod-shaped | +ve | + | - | - | - | + |
| **CB3.6** | Undulate, creamy, convex, shiny | Rod-shaped | −ve | + | + | - | - | + |
| **CB3.7** | Curled, creamy, convex, shiny | Rod-shaped | +ve | - | - | - | - | - |
| **CB3.8** | Curled, white, flat, dull | Rod-shaped | +ve | + | - | - | + | - |
| **CB3.9** | Undulate, white, pulvinate, dull | Rod-shaped | +ve | - | - | - | - | - |
| **CB3.10** | Curled, creamy, convex, shiny | Rod-shaped | +ve | - | - | - | - | - |
| **CB3.11** | Undulate, white, flat, shiny | Rod-shaped | +ve | - | - | - | + | - |
| **CB3.12** | Undulate, white, flat, shiny | Rod-shaped | +ve | + | - | - | + | - |
| **CB3.13** | Curled, creamy, flat, shiny | Rod-shaped | +ve | + | - | - | - | + |
| **CB3.14** | Undulate, white, pulvinate, dull | Rod-shaped | +ve | + | - | - | - | - |
| **CB3.15** | Curled, creamy, convex, shiny | Rod-shaped | +ve | + | - | - | - | - |
| **CB3.16** | Curled, white, pulvinate, shiny | Cocci-shaped | +ve | + | - | - | - | - |
| **CB3.17** | Undulate, white, convex, shiny | Rod-shaped | +ve | + | - | - | - | - |
| **CB3.18** | Undulate, creamy, convex, shiny | Rod-shaped | +ve | - | - | - | - | - |
| **CB3.19** | Entire, white, pulvinate, shiny | Rod-shaped | +ve | - | - | + | - | + |
| **CB3.20** | Undulate, white, pulvinate, shiny | Rod-shaped | +ve | - | - | - | - | - |
| **CB3.21** | Even, white, pulvinate, shiny | Rod-shaped | +ve | - | - | + | - | + |
| **CB3.22** | Even, white, convex, shiny | Rod-shaped | +ve | - | - | - | - | - |
| **CB3.23** | Even, white, convex, shiny | Rod-shaped | +ve | - | - | - | - | - |
| **CB3.24** | Curled, creamy, convex, shiny | Rod-shaped | +ve | - | - | - | - | - |
| **CB3.25** | Curled, white, convex, shiny | Rod-shaped | −ve | + | + | - | - | + |

**Abbreviation:** CB1- from Upper Core; CB2- Middle Core; CB3- Bottom Core, (+) sign indicates that the isolates showed a positive reaction, and (-) sign indicates that the isolates showed a negative reaction.

**Supplementary Table 2:** Carbohydrate fermentation test results of the selected isolates from different depths of the ice core of Kabru Glacier.

| **Upper Core (CB1) Isolates** | **D (-) Arabinose** | **L (+) Rhamnose** | **D (+) Galactose** | **D (+) Glucose** | **D (-) Mannitol** | **D (+) Cellobiose** |
| --- | --- | --- | --- | --- | --- | --- |
| CB1.1 | - | + | + | + | + | + |
| CB1.2 | - | - | + | + | - | + |
| CB1.6 | - | - | - | + | - | - |
| CB1.7 | + | + | + | + | + | + |
| CB1.11 | - | - | - | - | - | - |
| CB1.13 | - | - | - | - | - | - |
| CB1.15 | - | + | + | - | + | + |
| CB1. 18 | + | + | + | + | + | + |
| CB1.19 | + | + | + | + | - | - |
| CB1.23 | + | + | + | + | - | + |
| **Middle Core (CB2) Isolates** | **D (-) Arabinose** | **L (+) Rhamnose** | **D (+) Galactose** | **D (+) Glucose** | **D (-) Mannitol** | **D (+) Cellobiose** |
| CB2.1 | - | - | + | + | - | - |
| CB2.4 | - | - | + | + | - | - |
| CB2.5 | - | - | + | + | - | - |
| CB2.8 | - | - | - | + | - | - |
| CB2.17 | - | - | - | + | - | - |
| CB2.16 | - | - | - | + | - | - |
| CB2.19 | - | - | - | + | - | - |
| CB2.20 | - | - | - | + | - | - |
| CB2.21 | + | - | - | + | - | - |
| CB2.22 | - | - | - | + | - | - |
| **Bottom Core (CB3) Isolates** | **D (-) Arabinose** | **L (+) Rhamnose** | **D (+) Galactose** | **D (+) Glucose** | **D (-) Mannitol** | **D (+) Cellobiose** |
| CB3.4 | - | - | + | + | + | - |
| CB3.5 | + | + | + | + | + | + |
| CB3.6 | + | + | + | + | + | + |
| CB3.7 | - | - | - | - | - | - |
| CB3.12 | - | - | - | - | - | - |
| CB3.13 | - | - | - | - | - | - |
| CB3.15 | - | - | + | + | - | + |
| CB3.18 | - | - | - | - | - | - |
| CB3.19 | - | - | - | - | + | - |
| CB3.21 | - | - | - | + | - | + |

***Abbreviation:** ‘+’ indicates that the isolates were able to ferment the specific carbohydrate, and ‘-’ indicates that the isolates were unable to ferment the specific carbohydrate.

**Supplementary Table 3:** Screening of enzymes (amylase and protease) in the ice core isolates of Kabru Glacier.

|  | **Isolates** | **Amylase** | **Zone diameter (mm)** | **Protease** | **Zone diameter (mm)** |
| --- | --- | --- | --- | --- | --- |
| UPPER CORE | CB1.1 | × | NA | × | NA |
|  | CB1.2 | × | NA | × | NA |
|  | CB1.6 | × | NA | √ | 0.85 |
|  | CB1.7 | × | NA | × | NA |
|  | CB1.18 | √ | 1.00 | √ | 0.50 |
|  | CB1.19 | √ | 1.00 | √ | 0.62 |
|  | CB1.11 | √ | 1.30 | √ | 0.80 |
|  | CB1.13 | √ | 1.30 | √ | 0.65 |
|  | CB1.15 | √ | 1.20 | √ | 0.70 |
|  | CB1.23 | √ | 1.40 | √ | 0.50 |
| MIDDLE CORE | CB2.1 | × | NA | × | NA |
|  | CB2.4 | × | NA | √ | 1.20 |
|  | CB2.5 | × | NA | √ | 1.30 |
|  | CB2.8 | × | NA | × | NA |
|  | CB2.16 | × | NA | × | NA |
|  | CB2.17 | × | NA | √ | 0.95 |
|  | CB2.19 | √ | 1.05 | √ | 0.40 |
|  | CB2.20 | × | NA | √ | 0.60 |
|  | CB2.21 | × | NA | × | NA |
|  | CB2.22 | × | NA | √ | 0.70 |
| BOTTOM CORE | CB3.4 | × | NA | × | NA |
|  | CB3.5 | √ | 0.80 | × | NA |
|  | CB3.6 | × | NA | × | NA |
|  | CB3.7 | × | NA | × | NA |
|  | CB3.12 | √ | 1.10 | √ | 0.65 |
|  | CB3.13 | × | NA | √ | 0.95 |
|  | CB3.15 | √ | 0.40 | × | NA |
|  | CB3.18 | × | NA | × | NA |
|  | CB3.19 | × | NA | × | NA |
|  | CB3.21 | √ | 0.60 | √ | 0.10 |

**Abbreviation:** (√) sign indicates that the isolates gave positive reaction, (×) sign indicates that the isolates gave negative reaction against respective enzyme screening test, and NA is not applicable.

**Supplementary Table 4:** Growth pattern observed in the Kabru Glacier ice core isolates.

| **Kabru Ice Core** |  | **Temperature** | | | | | | **NaCl** | | | | | **pH** | | | | |
| --- | --- | --- | --- | --- | --- | --- | --- | --- | --- | --- | --- | --- | --- | --- | --- | --- | --- |
|  | **Isolates** | **4°C** | **10°C** | **15°C** | **20°C** | **37°C** | **50°C** | **0.2%** | **0.5%** | **1%** | **5%** | **10%** | **pH 3** | **pH 5** | **pH 7** | **pH 9** | **pH 11** |
| **Upper Core (CB1)** | **CB1.1** | - | + | ++++ | ++++ | ++ | - | + | +++ | ++++ | ++ | - | + | ++ | ++++ | +++ | - |
|  | **CB1.2** | - | + | ++++ | +++ | + | - | + | ++ | +++ | + | - | - | + | ++++ | +++ | - |
|  | **CB1.6** | - | ++ | ++++ | ++++ | +++ | - | + | +++ | ++++ | ++ | - | + | ++ | + | + | - |
|  | **CB1.7** | - | ++ | ++++ | ++++ | +++ | - | ++ | ++ | ++++ | +++ | - | + | ++ | ++++ | +++ | - |
|  | **CB1.11** | + | + | ++ | +++ | ++ | - | +++ | +++ | ++ | ++ | - | + | ++ | ++ | +++ | - |
|  | **CB1.13** | - | + | +++ | +++ | ++ | - | + | ++ | +++ | ++ | - | - | + | ++ | + | + |
|  | **CB1.15** | + | + | + | +++ | ++ | - | + | ++ | ++++ | ++ | - | + | ++ | ++++ | + | - |
|  | **CB1.18** | - | ++ | ++ | ++ | +++ | - | + | ++ | ++++ | +++ | - | - | ++ | ++++ | +++ | - |
|  | **CB1.19** | - | + | ++ | + | + | - | + | ++ | ++ | ++ | - | + | + | + | +++ | - |
|  | **CB1.23** | - | + | ++ | ++ | +++ | - | + | ++ | ++++ | ++ | - | + | + | +++ | + | - |
| **Middle Core (CB2)** | **CB2.1** | - | + | +++ | ++ | + | - | ++ | ++ | ++++ | ++ | - | - | + | ++ | +++ | - |
|  | **CB2.4** | + | +++ | ++++ | ++++ | + | - | + | + | ++ | + | - | + | ++ | +++ | ++ | - |
|  | **CB2.5** | + | + | ++++ | ++++ | + | - | + | + | ++ | + | - | - | ++ | ++++ | ++ | + |
|  | **CB2.8** | - | + | ++ | ++ | + | - | + | +++ | ++++ | ++ | - | + | ++++ | +++ | + | - |
|  | **CB2.16** | - | + | + | + | + | - | + | +++ | ++++ | ++ | - | - | ++ | ++++ | ++ | + |
|  | **CB2.17** | - | + | ++ | +++ | + | - | ++ | ++ | ++++ | +++ | - | - | ++ | ++++ | +++ | + |
|  | **CB2.19** | - | + | + | + | + | - | + | + | ++ | + | - | - | + | ++ | + | - |
|  | **CB2.20** | - | + | ++ | ++ | ++ | - | + | + | ++ | + | - | - | + | +++ | ++ | - |
|  | **CB2.21** | + | ++ | +++ | +++ | + | - | + | +++ | ++++ | + | - | - | ++ | +++ | + | - |
|  | **CB2.22** | - | + | ++ | ++ | + | - | ++ | ++ | +++ | + | - | - | ++ | ++++ | +++ | + |
| **Bottom Core (CB3)** | **CB3.4** | - | ++ | +++ | ++++ | ++++ | - | + | ++ | +++ | ++ | - | - | ++ | ++++ | +++ | + |
|  | **CB3.5** | - | ++ | ++++ | ++++ | + | - | + | ++ | ++ | + | - | - | + | ++ | + | - |
|  | **CB3.6** | - | ++ | ++++ | ++++ | + | - | + | + | ++ | + | - | + | + | ++++ | ++ | - |
|  | **CB3.7** | - | + | ++ | +++ | ++++ | - | ++ | ++ | ++++ | + | - | - | + | ++ | +++ | + |
|  | **CB3.12** | - | + | + | ++ | + | - | - | + | ++ | + | - | - | + | ++++ | +++ | + |
|  | **CB3.13** | - | + | + | ++ | + | - | ++ | ++ | ++++ | + | - | - | + | ++++ | +++ | + |
|  | **CB3.15** | - | ++ | ++ | ++ | + | - | + | ++ | +++ | ++ | - | - | ++ | ++++ | +++ | + |
|  | **CB3.18** | - | + | ++ | ++++ | ++++ | - | ++ | ++ | ++++ | ++ | - | - | ++ | ++ | ++ | - |
|  | **CB3.19** | + | + | ++ | ++ | + | - | + | + | +++ | ++ | - | - | + | ++++ | ++ | + |
|  | **CB3.21** | + | + | ++ | + | + | - | - | + | +++ | + | - | - | + | + | + | - |

**Abbreviation:** (+) sign indicate OD value ≤0.50, (++) indicate OD value >0.50 to ≤1.0, (+++) indicate OD value >1.0 to ≤1.50 and (++++) OD value >1.50.

**Supplementary Table 5:** Antibiotic susceptibility profile of bacterial isolates in the samples.

|  |  | Bacterial count of CB1 | | | Bacterial count of CB2 | | | Bacterial count of CB3 | | |
| --- | --- | --- | --- | --- | --- | --- | --- | --- | --- | --- |
| Antibiotic Classes | Antibiotic Subclasses | S | I | R | S | I | R | S | I | R |
| Quinolone | NA (30µg) | 5 | 2 | 3 | 6 | 3 | 1 | 4 | 3 | 3 |
| Aminoglycoside | GEN (10µg) | 10 | 0 | 0 | 10 | 0 | 0 | 9 | 0 | 1 |
|  | S (25µg) | 6 | 0 | 4 | 5 | 0 | 5 | 7 | 0 | 3 |
| Carbapenem | IMP (10µg) | 9 | 0 | 1 | 8 | 0 | 2 | 9 | 0 | 1 |
| Cephalosporin | CFM (5µg) | 4 | 0 | 6 | 2 | 0 | 8 | 3 | 0 | 7 |
| β-lactam | AMC (30µg) | 6 | 0 | 4 | 5 | 3 | 2 | 9 | 0 | 1 |
|  | AMP (10µg) | 6 | 0 | 4 | 2 | 0 | 8 | 9 | 0 | 1 |
|  | MET (10µg) | 5 | 0 | 5 | 4 | 0 | 6 | 6 | 0 | 4 |
| Tetracycline | DO (10µg) | 10 | 0 | 0 | 10 | 0 | 0 | 9 | 0 | 1 |
|  | TE (30µg) | 10 | 0 | 0 | 10 | 0 | 0 | 9 | 0 | 1 |
| Fluoroquinolone | OF (5µg) | 9 | 1 | 0 | 10 | 0 | 0 | 9 | 0 | 1 |
|  | CIP (5µg) | 0 | 0 | 1 | 10 | 0 | 0 | 8 | 1 | 1 |
| Glycopeptide | VA (30µg) | 10 | 0 | 0 | 6 | 0 | 4 | 9 | 0 | 1 |
| Chloramphenicol | C (30µg) | 10 | 0 | 0 | 8 | 0 | 2 | 9 | 0 | 1 |
| Azithromycin | AZM (15µg) | 10 | 0 | 0 | 8 | 0 | 2 | 9 | 0 | 1 |
|  | E (15µg) | 9 | 0 | 1 | 5 | 1 | 4 | 9 | 0 | 1 |

**Note****:** Sensitive represented by **S**, intermediate by **I**, and resistant by **R**. The isolates were tested against the following group of antibiotics: E = Erythromycin, CFM = Cefixime, TE = Tetracycline, C = Chloramphenicol, NA = Nalidixic acid, AMP = Ampicillin, IMP = Imipenem, AZM = Azithromycin, MET = Methicillin, CIP = Ciprofloxacin, VA = Vancomycin, S = Streptomycin, OF = Ofloxacin, GEN = Gentamycin, AMC = Amoxicillin, DO = Doxycycline hydrochloride (Bauer, 1966). *(n=10)

**Supplementary Table 6:** 16S rRNA gene Amplicon sequencing data of samples [Upper Core (UC), Middle Core (MC), Bottom Core (BC)] obtained using LotuS2 pipeline.

| **Glacier** | **Ice Core Sample** | **Reads processed** | **High quality reads** | **ASVs (denoised unique sequences)** | **Total reads in matrix** | **Sequence length** | **SRA Run Accession** |
| --- | --- | --- | --- | --- | --- | --- | --- |
| Kabru (UC) | CB1 | 290320 | 83096 | 160 | 125490 | 251 | SRR28976176 |
| Kabru (MC) | CB2 | 337439 | 90976 | 149 | 136857 | 251 | SRR28976175 |
| Kabru (BC) | CB3 | 322233 | 89468 | 188 | 138586 | 251 | SRR28976164 |

**Supplementary Table 7:** Relative abundance of genera in the glacier ice core sample.

| **Genera** | **Kabru Glacier** | | |
| --- | --- | --- | --- |
|  | **CB1** | **CB2** | **CB3** |
| ***Cronobacter*** | 20.20 | 20.50 | 19.30 |
| **Unclassified genera** | 19.90 | 19.10 | 18.40 |
| ***Staphylococcus*** | 16.10 | 17.70 | 15.70 |
| ***Bacillus*** | 13.80 | 16.10 | 12.91 |
| ***Mucilaginibacter*** | 10.70 | 9.39 | 10.70 |
| ***Escherichia*** | 8.59 | 7.57 | 8.91 |
| ***Klebsiella*** | 2.56 | 2.50 | 2.61 |
| ***Salmonella*** | 2.40 | 2.45 | 2.20 |
| ***Stenotrophomonas*** | 1.80 | 1.45 | 2.83 |
| ***Streptococcus*** | 0.59 | 0.86 | 1.05 |
| ***Enterococcus*** | 0.38 | 0.36 | 0.88 |
| ***Kocuria*** | 0.32 | 0.24 | 0.53 |
| ***Arcanobacterium*** | 0.28 | 0.23 | 0.29 |
| ***Acidisoma*** | 0.27 | 0.23 | 0.26 |
| ***Enterobacter*** | 0.27 | 0.22 | 0.26 |
| ***Corynebacterium*** | 0.25 | 0.14 | 0.48 |
| ***Pseudomonas*** | 0.25 | 0.16 | 0.39 |
| ***Weissella*** | 0.25 | 0.20 | 0.24 |
| ***Aliarcobacter*** | 0.22 | 0.16 | 0.42 |
| ***Clostridioides*** | 0.16 | 0.12 | 0.17 |
| ***Microcoleus*** | 0.15 | 0.10 | 0.27 |
| ***Micrococcus*** | 0.13 | 0.07 | 0.20 |
| ***Stenomitos*** | 0.10 | 0.08 | 0.22 |
| ***Paraburkholderia*** | 0.10 | 0.03 | 0.18 |
| ***Enterococcus_B*** | 0.06 | 0.05 | 0.12 |
| ***Paracoccus*** | 0.05 | 0.03 | 0.09 |
| ***Stutzerimonas*** | 0.04 | 0.03 | 0.10 |
| ***Arthrobacter*** | 0.04 | 0 | 0.05 |
| ***Acinetobacter*** | 0.02 | 0 | 0.06 |
| ***Phormidesmis*** | 0.02 | 0 | 0.04 |
| ***Modestobacter*** | 0.02 | 0 | 0.04 |
| ***Brevundimonas*** | 0 | 0 | 0.02 |
| ***Knoellia*** | 0 | 0 | 0.03 |
| ***Methylobacterium*** | 0 | 0 | 0.04 |
| ***Ochrobactrum*** | 0 | 0 | 0.03 |

**Note:** Values indicate relative abundance of genera in the sample and zero value indicates non-detection.

**Supplementary Table 8:** Alpha diversity indices of three ice core samples (CB1, CB2, CB3).

| **SAMPLE** | **OBSERVED RICHNESS** | **SHANNON_H** | **SIMPSON_1-D** |
| --- | --- | --- | --- |
| CB1 | 160 | 4.00 | 0.97 |
| CB2 | 149 | 3.85 | 0.96 |
| CB3 | 185 | 4.16 | 0.97 |
