# Supplementary material for: Exploring the bacterial diversity and its antibiotic resistance in Kabru Glacier ice cores, Sikkim Himalaya
Source: Front Microbiol. 2026 Jan 28;16:1672943. doi: 10.3389/fmicb.2025.1672943 (PMC12893349; doi:10.3389/fmicb.2025.1672943)
Supplement: Supplementary file 1 [file Data_Sheet_1.ZIP › Supplementary folder/STRevised Supplementary table caption.docx]

**Supplementary Table 1:** Morphological characteristics and biochemical properties of the bacterial isolates.

**Abbreviation:** CB1- from Upper Core; CB2- Middle Core; CB3- Bottom Core, (+) sign indicates that the isolates showed a positive reaction, and (-) sign indicates that the isolates showed a negative reaction.

**Supplementary Table 2:** Carbohydrate fermentation test results of the selected isolates from different depths of the ice core of Kabru Glacier.

***Abbreviation:** ‘+’ indicates that the isolates were able to ferment the specific carbohydrate, and ‘-’ indicates that the isolates were unable to ferment the specific carbohydrate.

**Supplementary Table 3:** Screening of enzymes (amylase and protease) in the ice core isolates of Kabru Glacier.

**Abbreviation:** (√) sign indicates that the isolates gave positive reaction, (×) sign indicates that the isolates gave negative reaction against respective enzyme screening test, and NA is not applicable.

**Supplementary Table 4:** Growth pattern observed in the Kabru Glacier ice core isolates.

**Abbreviation:** (+) sign indicate OD value ≤0.50, (++) indicate OD value >0.50 to ≤1.0, (+++) indicate OD value >1.0 to ≤1.50 and (++++) OD value >1.50.

**Supplementary Table 5:** Antibiotic susceptibility profile of bacterial isolates in the samples.

**Note****:** Sensitive, intermediate, and resistant are denoted by the letters S, I, and R, respectively. The isolates were tested against the following group of antibiotics: E = Erythromycin, CFM = Cefixime, TE = Tetracycline, C = chloramphenicol, NA = Nalidixic acid, AMP = Ampicillin, IMP = Imipenem, AZM = Azithromycin, MET = Methicillin, CIP = Ciprofloxacin, VA = Vancomycin, S = Streptomycin, OF = Ofloxacin, GEN = Gentamycin, AMC = Amoxicillin, DO = Doxycycline hydrochloride (Bauer, 1966). *(n=10)

**Supplementary Table 6:** 16S rRNA gene Amplicon sequencing data of samples [Upper Core (UC), Middle Core (MC), Bottom Core (BC)] obtained using LotuS2 pipeline.

**Supplementary Table 7:** Relative abundance of genera in the glacier ice core sample.

**Note:** Values indicate relative abundance of genera in the sample and zero value indicates non-detection.

**Supplementary Table 8:** Alpha diversity indices of three ice core samples (CB1, CB2, CB3).

.
