# Supplementary material for: Exploring the bacterial diversity and its antibiotic resistance in Kabru Glacier ice cores, Sikkim Himalaya
Source: Front Microbiol. 2026 Jan 28;16:1672943. doi: 10.3389/fmicb.2025.1672943 (PMC12893349; doi:10.3389/fmicb.2025.1672943)
Supplement: Supplementary file 1 [file Data_Sheet_1.ZIP › Supplementary folder/Supplementary Figure Caption.docx]

**Supplementary Fig.1:** Heatmap showing relative abundance of bacterial phyla obtained through culture-independent analysis, and the depth of the color in the cell indicates the relative percentage of phyla in each ice core sample. CB1 = Upper, CB2 = Middle, CB3 = Bottom.
