# Supplementary figures and images for: Exploring the bacterial diversity and its antibiotic resistance in Kabru Glacier ice cores, Sikkim Himalaya
Source: Front Microbiol. 2026 Jan 28;16:1672943. doi: 10.3389/fmicb.2025.1672943 (PMC12893349; doi:10.3389/fmicb.2025.1672943)

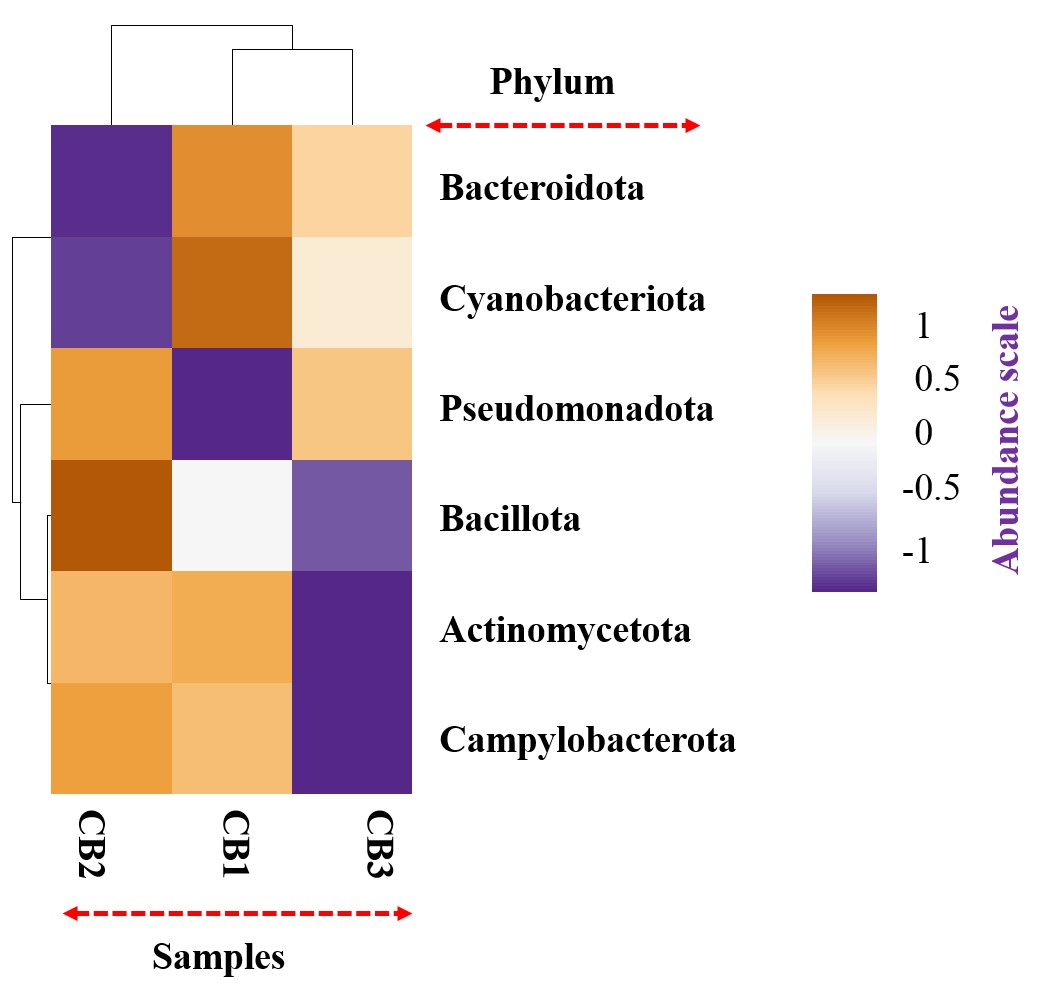

Supplement: Supplementary file 1 [file Data_Sheet_1.ZIP › Supplementary folder/Supplementary Fig. 1.jpeg]
